# Cyclotide Evolution: Insights from the Analyses of Their Precursor Sequences, Structures and Distribution in Violets (*Viola*)

**DOI:** 10.3389/fpls.2017.02058

**Published:** 2017-12-18

**Authors:** Sungkyu Park, Ki-Oug Yoo, Thomas Marcussen, Anders Backlund, Erik Jacobsson, K. Johan Rosengren, Inseok Doo, Ulf Göransson

**Affiliations:** ^1^Division of Pharmacognosy, Department of Medicinal Chemistry, Uppsala University, Uppsala, Sweden; ^2^Department of Biological Sciences, Kangwon National University, Chuncheon, South Korea; ^3^Department of Biosciences, Centre for Ecological and Evolutionary Synthesis, University of Oslo, Oslo, Norway; ^4^School of Biomedical Sciences, The University of Queensland, Brisbane, QLD, Australia; ^5^Biotech Research Team, Biotech Research Center of Dong-A Pharm Co Ltd., Seoul, South Korea

**Keywords:** cyclotide evolution, viola phylogeny, sequence signature, cyclotide precursor, neofunctionality, novel cyclotide, precursor domain

## Abstract

Cyclotides are a family of plant proteins that are characterized by a cyclic backbone and a knotted disulfide topology. Their cyclic cystine knot (CCK) motif makes them exceptionally resistant to thermal, chemical, and enzymatic degradation. By disrupting cell membranes, the cyclotides function as host defense peptides by exhibiting insecticidal, anthelmintic, antifouling, and molluscicidal activities. In this work, we provide the first insight into the evolution of this family of plant proteins by studying the Violaceae, in particular species of the genus *Viola*. We discovered 157 novel precursor sequences by the transcriptomic analysis of six *Viola* species: *V. albida* var. *takahashii, V. mandshurica, V. orientalis, V. verecunda, V. acuminata*, and *V. canadensis*. By combining these precursor sequences with the phylogenetic classification of *Viola*, we infer the distribution of cyclotides across 63% of the species in the genus (i.e., ~380 species). Using full precursor sequences from transcriptomes, we show an evolutionary link to the structural diversity of the cyclotides, and further classify the cyclotides by sequence signatures from the non-cyclotide domain. Also, transcriptomes were compared to cyclotide expression on a peptide level determined using liquid chromatography-mass spectrometry. Furthermore, the novel cyclotides discovered were associated with the emergence of new biological functions.

## Introduction

**Figure 1:**
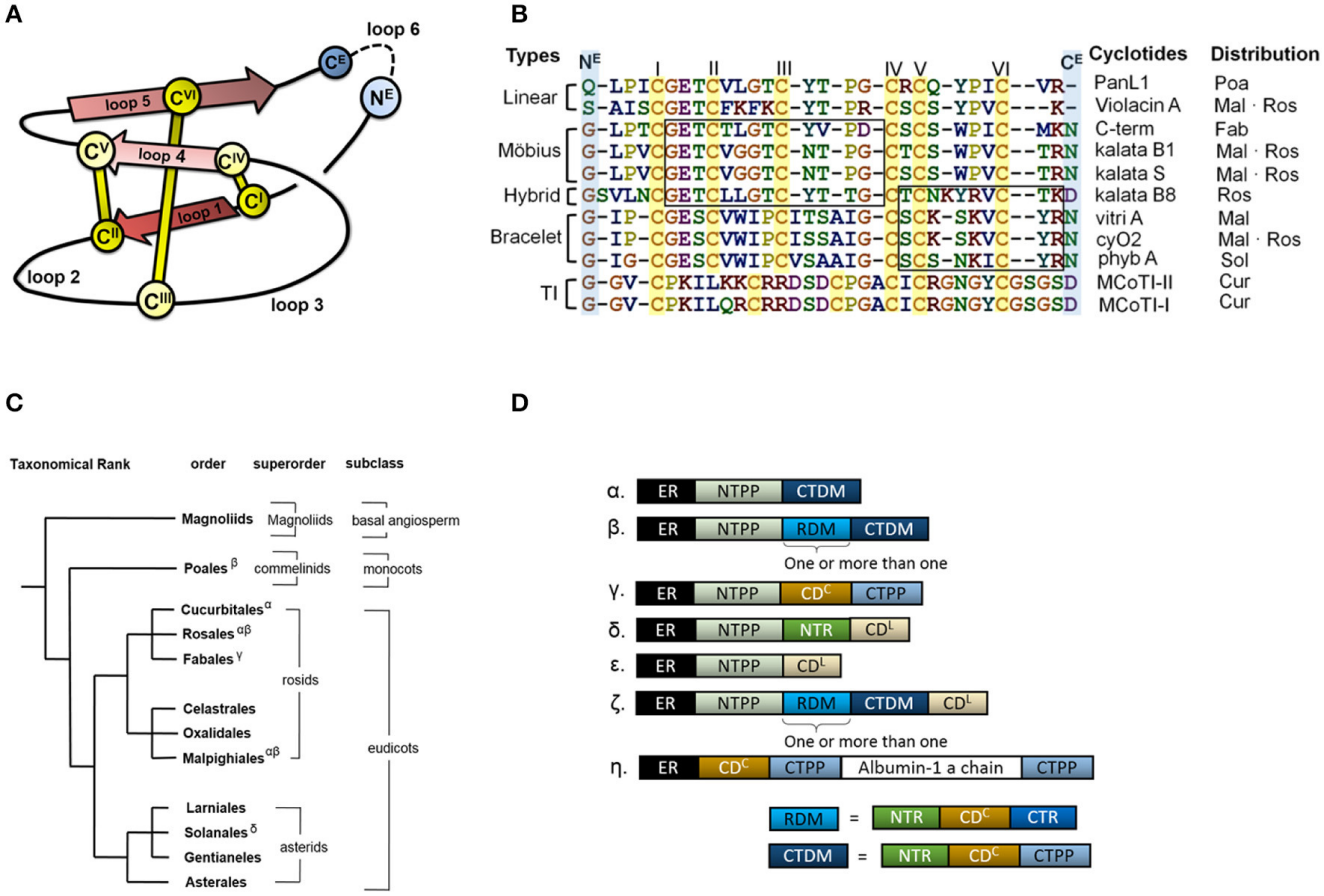
Cyclotide sequence, structure and distribution. **(A)** Schematic representation of the structure of a cyclotide and the CCK framework. The cyclization point between the C- and N-termini (N^E^ and C^E^, respectively) is highlighted, and the cysteine residues are labeled with Roman numerals. **(B)** Sequence alignment of representative cyclotides and their distribution in flowering plants. The cyclotides have been classified into five groups on the basis of their structural traits: Möbius cyclotides, bracelets, hybrids, linear cyclotides, and trypsin inhibitors (TI). Abbreviation code in plant orders: Poaceae (Poa), Malphigiales (Mal), Rosales (Ros), Fabales (Fab), Solanales (Sol) and Cucurbitales (Cur). The loops sharing sequence similarity are boxed. **(C)** Phylogeny of cyclotide-expressing flowering plants. To date, cyclotides have only been found in the eudicots. The family Violaceae belongs to the order of Malphigiales, Rubiaceae to Gentianales, and Cucurbitaceae to Cucurbitales. **(D)** Diversity of cyclotide precursor architectures. Precursor sequences consist of multiple domains: endoplasmic reticulum targeting signal (ER, black), N-terminal propeptide (NTPP, light blue), N-terminal repeat (NTR, green), cyclotide domain (CD), and C-terminal tail (CTR). Precursor architecture varies with both plant species and structural subfamily. Architecture types are labeled with Greek letters in **(D)**, and their distribution is shown in **(C)**. Oak1 (α) and Vok1 (β), precursors of kb1, are found in the Rubiaceae and Violaceae, respectively. Vok1 contains repeated domains. VocA (δ) and panitide pL1 (ε) are precursors of linear cyclotides and are found in the Violaceae and Poaceae, respectively. Precursors of these linear cyclotides do not contain a CTPP domain. TI subfamily precursors (ζ) contain both linear and cyclic cyclotides in the same sequence. Several architectures are often found in a single plant species. For example, *Viola* species contain VoK1 (α), VoC1 (β), and VocA (δ). In the precursor of Cter M, the cyclotide domain replaces the albumin-1b chain.

Cyclotides are proteins of ~30 amino acid residues that are characterized by the cyclic cystine knot (CCK) motif (Craik et al., 1999; Burman et al., 2014). The CCK motif consists of six conserved cysteines that form three disulfide bonds and a head to tail cyclic backbone (Figure 1A). The cyclotides have been classified into two main subfamilies, the Möbius and the bracelets, based on a single structural trait: the presence or absence of a conceptual 180° twist in the cyclic backbone caused by a conserved *cis*-Pro residue in loop 5 (Craik et al., 1999; Figure 1B). Aside from the CCK, two loops (defined as sequences between adjacent cysteines) have high sequence similarity between subfamilies (loop 1 and 4), whereas loops 2 and 3 are conserved only within individual subfamilies.

The discovery of other cyclotides has created a need for a more versatile classification system (Ireland et al., 2006; Nguyen et al., 2013; Ravipati et al., 2015). These varieties include so-called hybrid cyclotides that exhibit sequence characteristics of both the Möbius and bracelet subfamilies (Daly et al., 2006), as well as minor subfamily known as the trypsin inhibitors originating from gourd plants (Hernandez et al., 2000). They contain the CCK motif but do not otherwise exhibit any sequence similarity with the other subfamilies. In addition, linear cyclotide derivatives that exhibit sequence similarity with conventional cyclotides but lack their cyclic backbone have been reported (Ireland et al., 2006; Nguyen et al., 2013). The high sequence diversity of the cyclotides appears to be due to natural selection in angiosperms, the flowering plants, but little is known about the evolutionary mechanisms underpinning the corresponding selection processes or the evolutionary background of cyclotide diversity.

Cyclotides and the CCK motif have only been found in angiosperms, but proteins having one of their two defining structural motifs—cyclic peptides/proteins without the cystine-knot (Trabi and Craik, 2002; Arnison et al., 2013) or linear proteins with a cystine-knot (Zhu et al., 2003)—are found in a wide range of organisms across all kingdoms of life. In angiosperms, the occurrences of cyclotides differ between “basal” angiosperms, monocots and eudicots (Figure 1C): “linear cyclotides,” i.e., peptides that exhibit sequence similarity with cyclotides but lack their head-to-tail cyclic structure, are prevalent in both monocots and eudicots, but true cyclotides have been found only in eudicots (Mulvenna et al., 2006; Zhang et al., 2015a). However, neither linear nor true cyclotides have yet been found in the “basal” angiosperms. It has therefore been proposed that linear cyclotides are ancestral (more primitive) to the true cyclic cyclotides (Mulvenna et al., 2006; Gruber et al., 2008).

To date, cyclotides have been discovered in the eudicot families of Rubiaceae (coffee) (Gran, 1973a), Violaceae (violet family) (Schöpke et al., 1993; Claeson et al., 1998), Fabaceae (legume family) (Poth et al., 2011a), Solanaceae (potato) (Poth et al., 2012), and Cucurbitaceae (cucurbit) (Hernandez et al., 2000), as well as in the monocot family Poaceae (grass family) (Nguyen et al., 2013). Cyclotides appear to have functions in host defense because they exhibit insecticidal (Jennings et al., 2001), anthelmintic (Colgrave et al., 2008a), antifouling (Göransson et al., 2004), and molluscicidal (Plan et al., 2008) activities. In addition, native cyclotides have uterotonic (Gran, 1973b), anti-neurotensin (Witherup et al., 1994), antibacterial (Tam et al., 1999; Pränting et al., 2010), anti-HIV (Gustafson et al., 1994), anticancer (Lindholm et al., 2002), and immunosuppressive (Gründemann et al., 2012) activities. This plethora of activities and the stability of the CCK motif make them of interest for drug development (Northfield et al., 2014).

Many of these activities appear to be due to cyclotides' ability to interact with and disrupt biological membranes (Colgrave et al., 2008b; Simonsen et al., 2008; Burman et al., 2011; Henriques et al., 2011). The membrane disruption is mediated by physicochemical interactions between cyclotides and the lipid membrane, and is governed by the distribution of lipophilic and electrostatic properties over the molecular surfaces of the cyclotides. We recently developed a quantitative structure-activity relationship (QSAR) model for these interactions (Park et al., 2014). However, the relationships between cyclotide sequence diversity, evolutionary selection, and the functions of the cyclotides *in planta* remain unknown.

Cyclotides are expressed as precursor proteins, which undergo post-translational processing including enzymatic cleavage and subsequent cyclization (Jennings et al., 2001; Harris et al., 2015). The multi-domain architecture of these precursor proteins varies slightly between different types of cyclotides and plant families, but in sequential order from the N- to the C-terminus, they generally feature the following domains: an endoplasmic reticulum (ER) targeting signal, an N-terminal propeptide (NTPP), an N-terminal repeat (NTR), the cyclotide domain (CD), and finally a C-terminal tail (CTR) (Figure 1D). In some cases, the modular domains NTR, CD, and CTR are repeated more than once. The cyclotides have been suggested to co-evolve with asparaginyl endopeptidase (AEP) because of its suggested role in cyclization (Mylne et al., 2012). Moreover, the divergent evolution of cyclotides from ancestral albumin domains was suggested based on the architecture of cyclotide precursors found in the Fabaceae plant family (Nguyen et al., 2011; Poth et al., 2011b). However, the relationship between the precursor proteins' architecture and sequences and the evolutionary selection of cyclotides is still unknown.

To date, cyclotide and precursor sequences have been most extensively explored in the family Violaceae Batsch. (Malpighiales), and especially in the genus *Viola* L. (Burman et al., 2015). The Violaceae are a medium-sized family including ~1,100 species worldwide. The phylogeny of the Violaceae has recently been inferred from chloroplast and nuclear markers (Tokuoka, 2008; Wahlert et al., 2014), and its systematics has been revised accordingly; currently ~30 genera are accepted in nomenclature (Wahlert et al., 2014). *Viola* is the largest genus in the family, with 580–620 species, representing over 50% of all known species (Ballard et al., 1998; Yockteng et al., 2003; Marcussen et al., 2012; Wahlert et al., 2014). *Viola* is distributed all around the world in temperate regions and at high elevation habitats in the tropics. The genus is old (~30 million years) and comprises at least 16 extant lineages, referred to as sections, with a complex, reticulate phylogenetic history owing to allopolyploidy (Marcussen et al., 2012, 2015). The species included in this study belong to four north-temperate sections, the diploid sect. *Chamaemelanium* Ging. (*V. canadensis* L., *V. orientalis* W.Becker) and the three allotetraploid sections *Melanium* Ging. (*V. tricolor* L.), *Plagiostigma* Godr. (*V. albida* Palibin. var. *takahashii* (Nakai) Kitag., *V. mandshurica* W.Becker, *V. verecunda* A.Gray) and *Viola* (*V. acuminata* Ledeb.).

In the current study, we explore cyclotide evolution using an integrated approach, exploiting transcriptomics and peptidomics to analyze the sequences of cyclotide precursors and the expression of cyclotides in *Viola* in light of the phylogeny of the genus. In particular, full precursor sequences are used to obtain insights into the evolutionary history of the cyclotides, and we connect the evolution of new mature cyclotides with the emergence of new functions.

## Materials and methods

### Collection of violets

Violets were collected at the sites indicated in Table 1, and those sites are their natural habitats. When collected, the plant individuals were in adult stage; however, the exact ages of those plant individuals were not determined. The plant vouchers were deposited at the Kangwon National University herbarium; *Viola albida* var. *takahashii* (KWNU93021), *V. mandshurica* (KWNU93022), *V. orientalis* (KWNU93023), *V. verecunda* (KWNU93024), and *V. acuminata* (KWNU93025). All plant material was collected on September 23, 2014.

**Table 1:** The origins of the plant material examined in this work.

**Plants collected (Scientific name)**	**Locality**	**Geographic Coordinates and altitudes**
*V. albida* var. *takahashii*	Mt. Cheonma, Namyangju-si, Gyunggi-do, South Korea	N37°41′43.0″ E127°17′25.5″, 213 m
*V. mandshurica*	Kangwon National University Campus, Chuncheon-si, Gangwon-do, South Korea	N37°52′25.9″ E127°44′68.6″ 110 m
*V. orientalis*	Mt. Daeryong, Sumit area, Chuncheon-si, Gangwon-do, South Korea	N37°50′68.8″ E127°49′12.5″ 860 m
*V. verecunda*	Mt. Daeryong, Valley area, Chuncheon-si, Gangwon-do, South Korea	N37°50′03.5″ E127°49′80.7″ 415 m
*V. acuminata*	Mt. Oeum, Hoengsung-gun, Gangwon-do, South Korea	N37°36′34.9″ E127°56′12.9″ 619 m

### Sample collection, RNA isolation, and RNA sequencing

For the five *Viola* species—*V. albida* var. *takahashii, V. mandshurica, V. orientalis, V. verecunda*, and *V. acuminata*—, total RNA was sequenced by Next Generation Sequencing (NGS), outsourced to Macrogen Inc. (Seoul, South Korea). For each *Viola* species, the RNA sample was prepared from one plant individual; also, the plant tissues were pooled from all of the plant's major organs, i.e., the roots, stems, flowers, and leaves. The collected tissues were immediately frozen in liquid nitrogen, and directly extracted by RNeasy Plant Mini Kit (Qiagen, Hilden, Germany) according to the manufacturers' protocols. Quality and quantity of RNA were measured using an Agilent 2100 Bioanalyzer (Agilent Technologies, Santa Clara, CA, USA) with an RNA Integrity Number (RIN) and rRNA ratio. The measured RIN values (rRNA ratios) are: 6.9 (1.2) for *V. albida* var. *takahashii*, 7.4 (1.6) for *V. mandshurica*, 7.2 (0.2) for *V. orientalis*, 6.4 (0.9) for *V. verecunda* and 5.9 (0.7) for *V. acuminata*. For mRNA library preparation, a TruSeq RNA preparation kit was used according to the manufacturer's instructions (Illumina, San Diego, U.S.A.). Briefly, the poly-A containing mRNAs were purified using poly-T oligo-attached magnetic beads. The purified mRNAs were fragmented into short sequences by use of divalent cations. Using these short sequences as templates, the first-stranded cDNA was synthesized using random hexamers. A second-strand cDNA was then synthesized using DNA polymerase I and RNase H. The synthesized cDNA went through an end repair process, the addition of a single “A” base, and then ligation of the adapters. The PCR was performed to enrich the selected DNA sequences, and then those selected sequences were sequenced using Illumina HiSeq 2000 Sequencing System (Illumina, USA) that generates paired-end reads with 2 × 100 base pairs (bp) read length.

### Sequencing data analysis and assembly

FASTQC (version 0.11.3) was used to determine the quality of RNA sequencing data (www.bioinformatics.babraham.ac.uk/projects/fastqc/). Reads were cleaned using Trimmomatic (v. 0.32; Bolger et al., 2014), and the sequences with Phred score ≥33 and a minimum length of 36 bp were retained for assembly. *De novo* assembly of these processed reads was performed using the Trinity RNA-Seq assembly (release 17.07.2014; https://sourceforge.net/projects/trinityrnaseq/) with the default setup, which allows the identification of cyclotide-coding transcripts larger than 200 bp. Also, the reads were assembled separately for the individual species. We summarized the detailed information on the *de novo* transcriptome assembly for those individual species in Supplementary Table 1. The transcript abundance, Fragments Per Kilobase of transcript per Million mapped reads (FPKM; Trapnell et al., 2010), was calculated using Trinity based on RSEM algorithm (Li and Dewey, 2011).

### Identification of precursor sequences from transcriptome

The assembled transcriptomes were searched for similar sequences to the cyclotides from Cybase (cutoff date: 3.02.2014; http://www.cybase.org.au/) by the standalone NCBI-blast+ service (2.2.28) (tblastn, E-value cutoff: 50) in the Ugene software package (Okonechnikov et al., 2012). In parallel, a motif search was done using PROSITE for cyclotide precursor sequences [motifs PS60009 and PS60008 for Möbius and bracelet cyclotides, respectively (Sigrist et al., 2002)]. To assist sequence identification, the Fuzzpro service of EMBOSS (v. 5.0.0) (Rice et al., 2000) was used. The combined PROSITE and blast results were filtered to remove duplicates. The result file was further processed by manual inspection after clustal omega (v.1.2.0) alignment. In the manual inspection, we assumed that the sequence is a cyclotide precursor if the sequence contains the six conserved cysteines aligned with previously known cyclotides, and if the conserved N-terminal domain showed sequence similarity to known cyclotide precursors.

### Collection of precursor sequences

In total, 312 (= 157 + 155) precursor sequences were utilised in this study: (i) 157 (= 138 + 19) precursor sequences were identified and collected from transcriptome analyses conducted in the current study. Among them, 138 sequences were collected from the five *Viola* species (i.e., *Viola albida* var. *takahashii, V. mandshurica, V. orientalis, V. verecunda*, and *V. acuminata*) by the RNA sequencing in this study. The remaining 19 sequences were collected the transcriptomic data of *V. canadensis* obtained from the 1kp-project (www.onekp.com). (ii) Another 155 (= 126 + 29) precursor sequences were collected from recent published studies. Among them, the 126 sequences were collected from other seven *Viola* species (i.e., *V. baoshanensis, V. odorata, V. uliginosa, V. adunca, V. tricolor, V. biflora*, and *V. pinetorum*), and the 29 sequences from other Violaceae genera, i.e., *Gloeospermum, Melicytus*, and *Pigea*. The name of *Viola* species where the precursor sequences collected from and the corresponding references are listed in the Table 2, and the sequence alignment of those 312 precursors are recorded in the Supplementary Data 1.

**Table 2:** Violaceae species sampled for the analysis of precursor sequences.

**Genus**	**Section**	**Species**
*Viola*	*Plagiostigma*	***V. mandshurica*** **W.Becker** [37]^a^, ***V. albida*** **Palib. var**. ***takahashii*** **(Nakai) Kitag**. [29]^a^, ***V. verecunda*** **A.Gray** [23]^a^, *V. baoshanensis* W.S.Shu, W.Liu & C.Y.Lan [44]^b^
	*Viola*	*V. odorata* L. [5]^c^, *V. uliginosa* Bess. [18]^d^ ***V. acuminata*** **Ledeb**. [23]^a^ *V. adunca* Sm. [2]^e^
	*Melanium*	*V. tricolor* L. [49]^f^
	*Chamaemelanium*	***V. orientalis*** **W.Becker** [26]^a^, *V. biflora* L. [6]^g^, ***V. canadensis*** **L**. [19]^h^*, V. pinetorum* Greene [2]^e^
*Gloeospermum*		*G. blakeanum* (Standl.) Hekking. [5]^i^, *G. pauciflorum* Hekking. [4]^i^
*Melicytus*		*M. ramiflorus* J.R.Forst. & G.Forst. [14]^j^
*Pigea*		*P. floribunda* Lindl. (= *Hybanthus floribundus* (Lindl.) F.Muell.) [6]^k^

*The numbers in parentheses after the species name show the number of precursor sequences derived from that species. Species names marked in bold were used for transcriptomic assay of the current study. Sections are indicated within Viola. Within sect. Plagiostigma, V. mandshurica, and V. albida var. takahashii belong in subsect. Patellares (Boiss.) Rouy & Foucaud, V. verecunda in subsect. Bilobatae (W.Becker) W.Becker and V. baoshanensis in subsect. Diffusae (W.Becker) Chang. Within sect. Viola, V. odorata belongs to subsect. Viola and V. acuminata, V. adunca, and V. uliginosa in subsect. Rostratae (W.Becker) W.Becker. No subsections are applicable to sect. Chamaemelanium*.

a *Transcriptomes from the current study, V. mandshurica, V. albida var. takahashii, V. verecunda, V. acuminata and V. orientalis*;

b *V. baoshanensis (Zhang et al., 2009, 2015b)*;

c *V. odorata (Dutton et al., 2004; Ireland et al., 2006)*;

d *V. uliginosa (Slazak et al., 2015)*;

e *V. pinetorum and V. adunca (Kaas and Craik, 2010)*;

f *V. tricolor (Mulvenna et al., 2005; Hellinger et al., 2015)*;

g *V. biflora (Herrmann et al., 2008)*;

h *Transcriptome data for V. canadensis were obtained from the 1kp project (www.onekp.com), and the cyclotide precursor sequences were determined in this work*;

i *G. blakeanum and G. pauciflorum (Burman et al., 2010)*;

j *M. ramiflorus (Trabi et al., 2009)*;

k *P. floribunda (as H. floribundus) (Simonsen et al., 2005)*.

### Nomenclature of cyclotides and precursors

Each cyclotide sequence was assigned a tripartite name. The first part is derived from the Latin binomial of the plant in which the corresponding precursor was found, the third part specifies the molecular species of the cyclotide, and the second part specifies the rank of the cyclotide among all the cyclotides having that particular molecular species that were identified in that particular plant species (Supplementary Table 2). The Latin binomials of the violet species considered in this work are *Viola albida* var. *takahashii* (valta), *Viola mandshurica* (viman)*, Viola orientalis* (vorie)*, Viola verecunda* (viver), and *Viola acuminata* (vacum). Thus, the cyclotide named *vacum2-HS4* is the second cyclotide of the HS4 molecular species derived from *V. acuminata*. The molecular species are named after their NTR signature sequences, i.e., the identities of the two residues at the consensus positions 11 and 12. The precursor sequences were also assigned tripartite names in a similar way to the cyclotides: the first part of the name is derived from the Latin binomial of the species in which the sequence was discovered, the second specifies the numerical rank of the sequence (which is independent of that assigned to the cyclotides), and the third specifies the molecular species of the precursor. Any cyclotide or precursor that had been named in an earlier publication was assigned the same name in this work. However, for previously unnamed precursors, we added the prefix “prc” to the tripartite name to help readers distinguish between precursor and cyclotide sequences. Thus, prc-viul A is the name of the *pr*e*c*ursor sequence of Viul A from *Viola uliginosa* Bess.

### Sequence alignment for phylogenetic analysis

The cyclotide precursor sequences from transcriptome were aligned before the phylogenetic analysis i.e., construction of Bayesian phylogenetic tree, maximum parsimony and splits networks. A total of 92 precursor sequences (two sequences from each of the 46 molecular species) were prepared as DNA sequence for the sequence alignment, and the sequences include only three domains of the precursor, i.e., NTPP, NTR, and cyclotide domains. To guide the alignment of DNA sequences of the precursors, they were translated into protein sequences and aligned independently for each molecular species using Clustal in BioEdit v.7.2.5 (Hall, 1999), and those alignments were in turn combined and realigned (i.e., keeping the indel positions from the alignment of each of the molecular species). The resulting protein-guided alignment of nucleotide sequences was then subject to manual adjustments within reading frames. The aligned DNA sequences are shown in the Supplementary Data 2.

### The construction of Bayesian phylogenetic tree

Nucleotide substitution model was selected based on the AICc criterion using JModelTest v.2.1.10 (Guindon and Gascuel, 2003; Darriba et al., 2012). A Bayesian phylogenetic analysis was conducted in BEAST v.1.7.4 (Drummond and Rambaut, 2007; Drummond et al., 2012). The analysis file was set up in BEAUti (part of the BEAST package) with the following priors: GTR+G as substitution model with empirical frequencies and four gamma categories, a lognormal relaxed clock prior with rate set to 1.0, and a Yule tree prior (birth-only process). Two MCMC chains were run for 50 million generations each, using a BEAGLE library, and parameters logged every 10,000 generations. We checked the two chains for proper mixing and convergence (i.e., ESS >500) in Tracer v.1.6.0 (http://tree.bio.ed.ac.uk/software/tracer/), removed a visually determined burn-in of 1 million generations from each and merged the two chains in LogCombiner v.1.7.4 (part of the BEAST package), and summarized the data in a maximum credibility tree with mean node heights using TreeAnnotator v.1.7.4 (part of the BEAST package). The resulting tree was visualized and edited in FigTree v.1.4.3 (http://tree.bio.ed.ac.uk/software/tracer/). The input file for the BEAST and the resulting phylogenetic tree are found in the Supplementary Data 3, 4, respectively.

### The construction of splits networks

In order to visualize the data, neighbor splits-networks of uncorrected P distance were produced, both for the nucleotide alignment and for the translated alignment, using SplitsTree (Huson and Bryant, 2006).

### Extraction of plant material

Between 250 and 500 mg of dried plant material was homogenized and incubated overnight in 6 ml of 60% acetonitrile in water containing 0.05% triflouoroacetic acid (TFA) to extract cyclotides. Extracts were lyophilized and then dissolved in 2.5 ml of solvent A (Milli-Q H_2_O with 0.05% TFA). Redissolved extracts were then subjected to gel filtration using PD-10 columns (GE Healthecare) according to the manufacturer's instructions to remove small molecules. The high molecular weight fractions were collected, lyophilized and then dissolved in solvent A to a concentration proportional to the original amount of extracted material (2 μl/1 mg) for LC-MS analysis.

### LC-MS

The samples were analyzed using ultra performance liquid chromatography coupled to quadrupole time-of-flight mass spectrometry (nanoAcquity UPLC/QTof Micro; Waters, Milford, MA). Samples were eluted using a gradient of acetonitrile (1 to 90% over 50 min) containing 0.1% formic acid. A nanoLC column (Waters BEH, 75 μm (i.d) × 150 nm) operated at 0.3 μl/min flow rate was used. The capillary temperature was set at 220°C and the spray voltage at 4 kV. The mass-to-charge (m/z) range was set from 1,000 to 2,000. LC-MS chromatogram and MS spectra were analyzed with the help of MassLyxn V4.1 (Waters, Milford, MA).

### Reduction and alkylation

Dried peptide extracts were reduced in a buffer containing 0.05 M Tris-HCl, pH 8.3, 4.2 M guanidine-HCl, and 8 mM DTT. The extraction solutions were incubated at 37°C for 2 h in the dark, followed after O_2_ removal with Nitrogen gas. The reduced peptides were then further alkylated in a buffer containing 0.2 M Tris-HCl, pH 8.3, and 200 mM iodoacetamide for 1 h.

### Identification of cyclotides from the plant extracts

**Figure 2:**
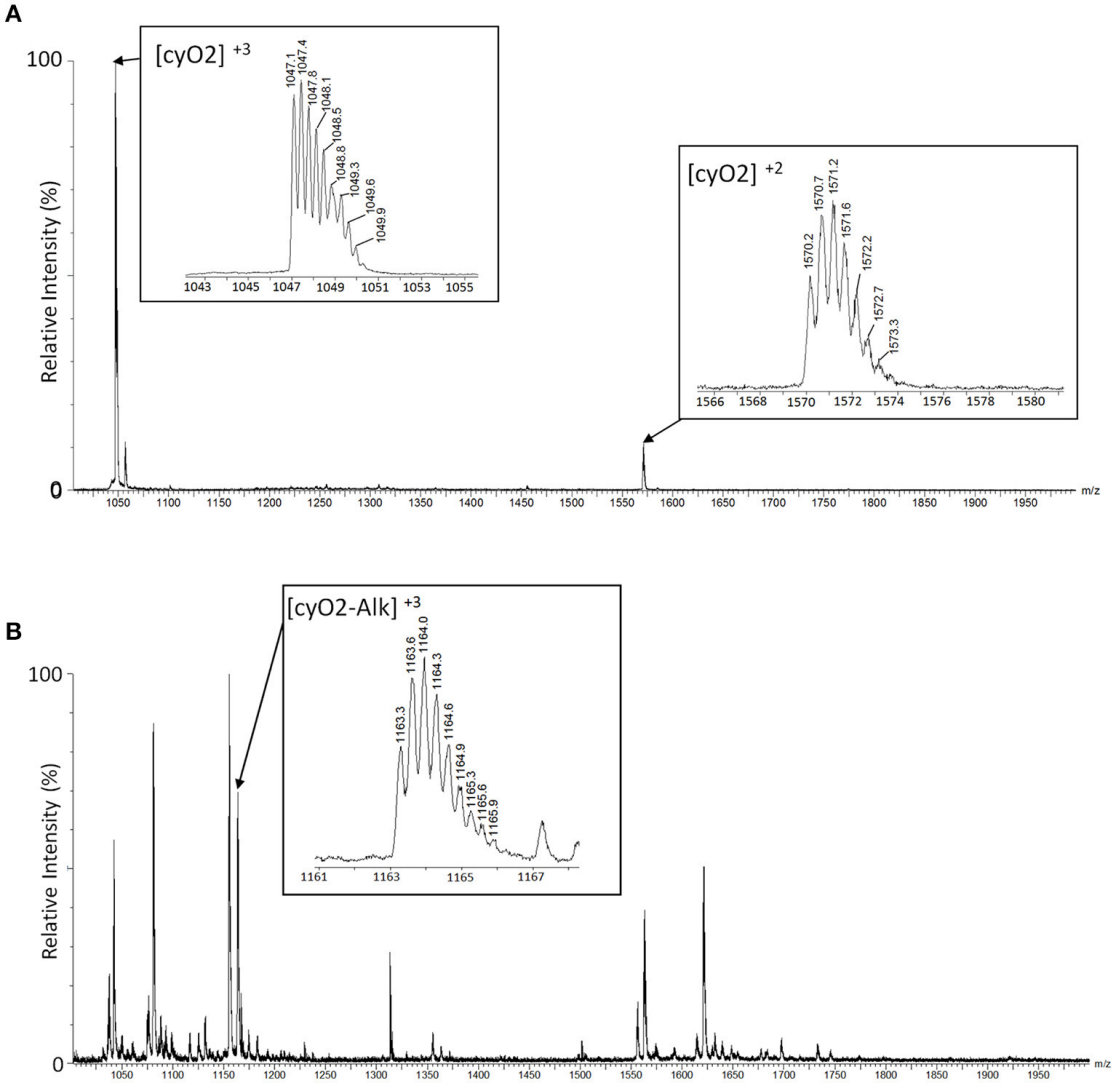
Identification of cyclotides by alkylated mass changes on LC-MS. Mass spectra of a cyclotide (cyO2) are shown with a comparison between the mass spectra of the plant extract before alkylation **(A)** and after alkylation **(B)**. **(A)** We identified the mass spectra of the cyclotide-like using the mass distance on their isotopic peaks. The triple-charged monoisotopic mass of the cyO2 (marked as [cyO2]^+3^ in the box) is observed as 1047.1Da, and the double-charged monoisotopic mass ([cyO2]^+2^) is observed as 1570.2Da. In each mass spectrum, the distances between isotopic peaks are observed with ~0.3Da for [cyO2]^+3^, and 0.5Da for [cyO2]^+2^. The monoisotopic mass of the cyO2 is 3138.4Da (calculated) and 3138.3Da (observed), and the mass difference is 0.1Da. **(B)** The monoisotopic mass of the cyO2 after the alkylation reaction is 3486.6Da (calculated) and 3486.9Da (observed), and the mass difference is 0.3Da. The calculated mass is based on the triple-charged monoisotopic mass of cyO2 (1163.3Da), and its related mass spectrum is shown in the box marked [cyO2-Alk]^+3^.

On the original LC-MS chromatogram, all chromatographic peaks were manually investigated if they include a cyclotide-like mass spectrum. We assumed that peaks stemmed from cyclotide-like substances if their masses fell within the range of 2,700–3,300 Da, as deconvoluted from their doubly- and triply charged ions. Then, the presence of three disulfide bonds was used to support their identification as cyclotides: if peaks in the extract showed an increase in mass by 348.18 Da, we considered that the peak stemmed from a true cyclotide (Figure 2).

### Matching transcriptomic data to cyclotides on the protein-level

Presence of possible cyclotides identified in precursor sequences from transcriptome analyses was determined as follows: putative cyclotide sequences were listed and their monoisotopic mass were calculated. The listed cyclotide sequences were comprised of N-terminal residues ranging from [−3, 2] on the consensus position of their precursor sequences. The C-terminal residues were found differently between cyclic cyclotides and linear cyclotides, i.e., the highly conserved N/D residue located at loop 6 for cyclic cyclotides, and the residue located next to stop codon in their mRNA sequences for linear cyclotides. These calculated monoisotopic mass were then compared to the observed monoisotopic mass from the identified cyclotides in plant extracts using LC-MS. We regarded that the transcriptomic cyclotides were expressed in the protein-level if the difference of the monoisotopic mass <0.40 Da.

### Determination of cyclotides' abundance levels in the proteome

For each cyclotide-like substance, the chromatographic peaks were identified together with their own retention time from the original LC-MS chromatogram. These chromatographic peaks were further investigated in relation with their mass spectral peaks to estimate the signal intensity (SI) of the cyclotide-like substance. The SI is estimated as a summed signal intensity of triple-charged mass spectral peaks. According to the SI, we assigned the cyclotide abundance into three levels, i.e., the abundance level is: low if SI <250, high if SI > 1,000, and medium if 250 < SI < 1,000.

### The calculation of molecular descriptors

The physicochemical properties of selected cyclotides (Park et al., 2014), i.e., the total lipophilicity and the exposure ratio, were calculated using scientific vector language (SVL), implemented in MOE 2012 (Chemical Computing Group Inc., Montreal, Canada).

### Accession numbers

The accession numbers of Sequence Read Archive (SRA) and Transcriptome Shortgun Assembly (TSA) for transcriptome of the five *Viola* species are: *V. acuminata* (TSA: GFWD00000000, SRA: SRR5320546), *V. orientalis* (TSA: GFXR00000000, SRA: SRR5322130), *V. albida* var. *takahashii* (TSA: GFWC00000000, SRA: SRR5320531), *V. mandshurica* (TSA: GFWG00000000, SRA: SRR5320533), and *V. verecunda* (TSA: GFWF00000000, SRA: SRR5322180).

## Result and discussion

The family Violaceae and the genus *Viola* are an excellent system for studying the evolution of cyclotides. All investigated species express large numbers of these proteins (Burman et al., 2015) as well as their precursor sequences. Furthermore, the species-level phylogeny of *Viola* (Marcussen et al., 2012, 2015) and chloroplast phylogeny of Violaceae (Wahlert et al., 2014) are well-understood as a result of recent studies. In the current study, 312 (= 157 + 155) sequences were analyzed in total to investigate the evolution of cyclotides. Among them, 157 precursor sequences were discovered from the transcriptomes of the six *Viola* species, i.e., *V. albida* var. *takahashii, V. mandshurica, V. orientalis, V. verecunda, V. acuminata*, and *V. canadensis* (listed in the Supplementary Table 3). Those discovered 157 sequences were pooled with the other 155 precursor sequences recently found from the other *Viola* species (i.e., *V. baoshanensis, V. odorata, V. uliginosa, V. adunca, V. tricolor, V. biflora*, and *V. pinetorum*) and from other Violaceae genera (i.e., *Gloeospermum, Melicytus*, and *Pigea*).

### The sequence signature of the prodomain can be used for the classification of cyclotides

The structural classification of cyclotides into Möbius cyclotides, bracelets, and hybrids thereof based on the structure and sequence of the mature cyclotide is here replaced by a classification based on the sequences of the cyclotide precursors, including both the prodomain (i.e., the NTPP and NTR domains) and the cyclotide domain. We focused on these three domains (i.e., the NTPP, NTR, and cyclotide domains) because they are present in all of the precursors that have been completely sequenced. The ER domain was not included in the analysis because ER domain sequences often vary strongly with the quality of the sequencing data. Other domains (i.e., the CTR domain and repeats of the NTR and cyclotide domains) are not present in all precursors, and were therefore also excluded.

**Figure 3:**
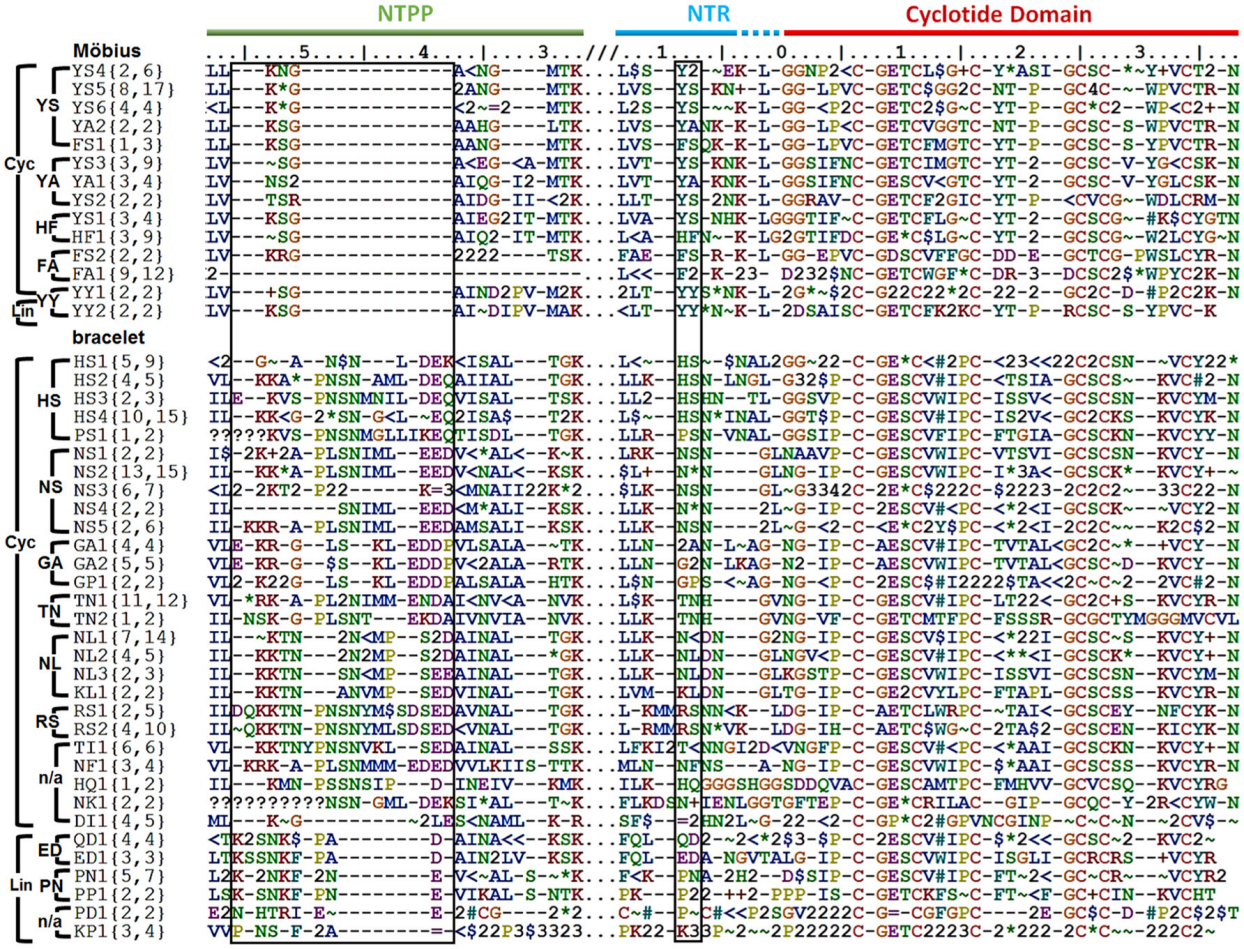
Classification of cyclotide precursors based on the sequence signature. Consensus sequences of the molecular species identified in this work. Based on the sequence signatures in the prodomain, 249 cyclotide precursors were classified into the Möbius and bracelet lineages, 13 molecular series and 46 molecular species (14 Möbius and 32 bracelet species). Species are named for their signature sequences, and numbers within brackets show the numbers of complete and total (complete and partial) precursor sequences for each species. The left box shows the NTPP indel region [−56, −38], which contains defining sequence gaps at [−56, −54], and [−50, −38] in the Möbius lineage. Within a lineage, specific prodomain sequence-traits are associated with different structural subfamilies. In the Möbius lineage, the sequence (Y^∧^/F^∧^/H)^−9^-(S^∧^/A/Y)^−8^ at position [−9, −8] is associated with cyclic cyclotide precursors (where “^∧^” indicates high occurrence of the labeled residue), while Y^−9^-Y^−8^ is associated with linear cyclotide precursors. Precursors of structural hybrids exhibit have an insertion at [−32, −31], whereas the archetypical Möbius precursors have a deletion at this position. In the bracelet lineage, the sequences (H^∧^/N^∧^/S/T/G/K/P)^−9^-(L/N/S/F/A)^−8^ and (Q/E/P/K)^−9^-(D/N)^−8^ are associated with cyclic and linear precursors, respectively. In addition, linear precursors contain a deletion that is flanked by two defining insertions, (P^∧^/A/L)^−49^-(N^∧^/A)^−48^ and (D/E)^−39^, in the NTPP at [−49, −48] and [−39]. Conserved amino acids are indicated by their one-letter codes, and variable residues are represented by symbols indicating the physicochemical properties of their side chains: hydrophilic (~), hydrophobic ($), flexible (G), rigid (P), disulfide-forming (C) and indels (–). Hydrophilic residues are further divided into positively charged (+), negatively charged (=) and uncharged hydrophilic (^*^); hydrophobics ($) are divided into aromatic (#) and alkyl (<) groups. Numbers are used as consensus symbols for multiple physicochemical categories.

Precursors were classified by their *sequence signatures*, i.e., the patterns of insertions and deletions (indels) and conserved sequences in the prodomains (NTPP and NTR). The N-terminal cleavage site of the cyclotide domain was defined as position 0, and a large indel region was detected upstream in the NTPP at positions [−56, −38] (Figure 3). Within this indel, the insertion region features sequence variations with minor gaps, and the deletion region has definite sequence gaps [−56, −54], [−50, −38]. Interestingly, the insertions coincide with cyclotide domains of archetypical bracelet cyclotides (e.g., cycloviolacin O2), while deletions coincide with cyclotide domains of archetypical Möbius cyclotides (e.g., kalata B1). Based on these observations, we suggest that these indels in the NTPP domain can be used as a criterion for classifying precursors into the Möbius and bracelet *lineages*. The term lineage is used instead of subfamily in order to avoid confusion with the structural classification of the cyclotides.

Within each lineage, precursors can be further divided by analogy to their structural classifications. That is to say, it is possible to identify sequence traits associated with linear and cyclic cyclotides within each lineage, and sequence traits associated with archetypical and hybrid subfamilies within the cyclic cyclotides of the Möbius lineage. Signature sequences of residues [−56, −38] were used to subdivide the bracelets, and residues [−32, −31] for the Möbius lineages, together with residues [−9, −8] of the NTR. Within the bracelet lineage, the sequence signatures associated with linear and cyclic cyclotide precursors differ in two ways. First, linear cyclotide precursors contain a signature sequence in the NTPP domain defined by two sequence insertions at [−49, −48] and [−39], together with a sequence deletion between those two sequence insertions. The signature sequences at these two locations are (P^∧^/A/L)^−49^-(N^∧^/A)^−48^ and (D/E)^−39^, where the symbol ^∧^ denotes a residue that occurs most frequently at the indicated position. Conversely, precursors of cyclic peptides have variable sequences with minor gaps in the [−47, −40] region. Second, whereas positions [−9, −8] in the NTR of precursors of linear cyclotides are conserved, having the signature sequence (Q/E/P/K)^−9^-(D/N)^−8^, the precursors of cyclic cyclotides show higher diversity, having sequences of (H^∧^/N^∧^/S/T/G/K/P)^−9^-(L/N/S/F/A)^−8^. Different signature sequences were also identified between the three structural subfamilies of the Möbius lineage, i.e., linear, hybrid and archetypical Möbius cyclotides. First, while precursors of both linear and hybrid cyclotides contain a sequence insertion in the NTPP domain at [−32, −31], precursors of archetypical Möbius cyclotides contain a deletion at this position. Second, while the sequence at [−9, −8] in the NTR domain of linear cyclotides is highly conserved (Y^−9^-Y^−8^), it is more variable in the precursors of cyclic cyclotides (Y^∧^/F^∧^/H)^−9^-(S^∧^/A/Y)^−8^.

On the basis of these findings, i.e., the combined sequence signatures of the NTPP and NTR domains and the conservation of residues with similar physicochemical properties in the cyclotide domain, two new classification orders were defined and used to classify the members of each lineage. These new orders were termed the molecular species and molecular series. Precursors with the same sequence signatures in both the NTPP and NTR domains are assigned to the same molecular species, while precursors that only share signature sequences in the NTR domain are assigned to the same molecular series.

Of 283 precursor sequences, 80 were classified into the Möbius lineage and 181 into the bracelet lineage (Figure 3; see Supplementary Figure 1). Of these 261 sequences, 249 were classified into 13 molecular series and 46 molecular species. Thus, 78 sequences representing the Möbius lineage were classified into five molecular series and 14 molecular species, while 171 sequences representing the bracelet lineage were classified into 8 molecular series and 32 molecular species. The remaining precursor sequences (34 of the initially examined 283, or 12.0% of the total) could not be grouped with any other sequences, and not be classified with molecular species (Supplementary Figure 2). Most of these sequences (31/283, or 10.9% of the total) were either partial sequences or lone unique sequences. Only three of all the precursor sequences (3/283) did not exhibit features enabling their classification into a given lineage on the basis of their NTPP domain sequences.

**Figure 4:**
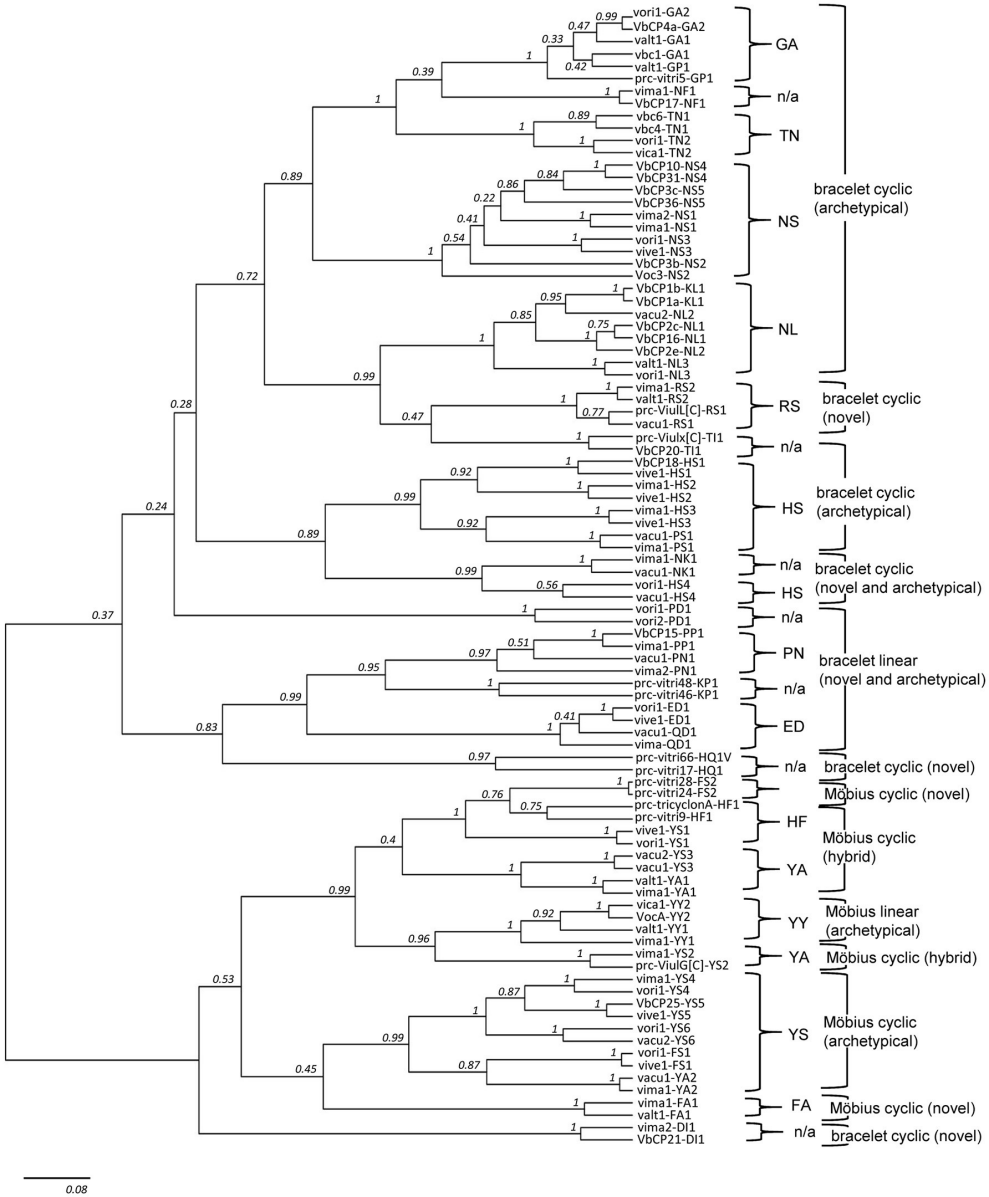
Phylogenetic tree of cyclotide precursors. The tree contains 92 precursor sequences in total, with two representative sequences from each of the 46 molecular species. Except from the nine molecular species (i.e., GA2, GP1, NS2, NS5, NL2, HS4, PN1, QD1, and YY1), all other 37 molecular species (80%, 37/46) are grouped as one monophyletic clade (posterior probability (*pp*) ≥ 0.75). Also, except from four molecular series (i.e., FA, HF, HS, and YA), the molecular species belong to other nine molecular series are monophyletic (*pp* ≥ 0.97). In the phylogenetic tree, DI1 is a single exception from the lineage classification based on sequence signature. DI1 is grouped into Möbius lineage in the phylogenetic tree (*pp* ≥ 0.70), even though it is classified as bracelet lineage based on the sequence signature.

Informal hierarchical ranks were then assigned to this system for classifying precursors, with lineage being the highest ranking classification, followed by molecular series and then molecular species. We further investigated the evolutionary relevance of this classification system by performing the phylogenetic analysis using the full DNA precursor sequences (Figure 4). We assumed that if the sequence signatures are highly conserved in the course of the evolution, the phylogenetic relationship of the precursor sequences has the hierarchical ranks as classified based on the sequence signatures. In this evaluation, we adopted a Bayesian phylogenetic approach using BEAST, because substitution model-based methods are in most cases better than Maximum Parsimony or distance-based methods at handling homoplasy, which is prominent among the cyclotide precursor sequences (Splits network shown in Supplementary Figure 3), and because BEAST sets a prior on the branch lengths that is particularly suitable also for analysis of short DNA sequences, such as cyclotide precursors.

The two lower hierarchical ranks (i.e., molecular species and series) are consistently recovered in the phylogeny, but not the upper hierarchical rank (i.e., molecular lineage), as indicated by the low posterior support values at the base of the phylogeny (Figure 4). Most of precursor sequences belong to the same molecular species are grouped as one monophyletic clade (80%, 74/92) or to the different clades but more closely than the precursor sequences derived from the different molecular series. Among 13 molecular series, the molecular species belong to the four molecular series (i.e., HF, YA, FA, and HS) are not monophyletic. Only one molecular species (DI1) is exceptionally grouped into Möbius lineage in the phylogenetic tree, even though the DI1 is classified as the bracelet lineage based on the sequence signature. In the phylogenetic analysis, we selected a total of 92 precursor sequences by the random selection of two sequences from each of the 46 molecular species. We assumed that such precursor selection would be enough to show a phylogenetic support for the classification system based on the sequence signature approach, because we randomly selected two sequences from each molecular species, and these sequences were mostly paired or grouped into monophyletic clades in accordance with the classification at the level of molecular species and series.

### The distribution of cyclotide precursors reflects the phylogeny of the genus *Viola*

Precursor sequences were compared to the established phylogenetic relationships between the four infrageneric sections of *Viola* (i.e., sects. *Melanium, Plagiostigma, Viola*, and *Chamaemelanium*), and between the genus *Viola* and the other Violaceae genera. The presence of a molecular species in different *Viola* taxa, such as species or sections, indicates that the molecular species evolved prior to the most recent common ancestor of these taxa. However, such inferences in *Viola* are complicated by the network-like phylogeny of the genus, owing to repeated ancient events of allopolyploidy (Marcussen et al., 2015). Hence, out of four sections in the current analysis, three (i.e., sects. *Melanium, Plagiostigma, Viola*) are allotetraploids originated by independent hybridizations between the same two parental lineages MELVIO and CHAM around 15 million years ago. The last section, sect. *Chamaemelanium*, is diploid and descends from the CHAM lineage. Because the parental MELVIO lineage is now extinct, it is impossible to infer with certainty the distribution of the molecular species in the common ancestor of these parental lineages, i.e., the CHAM and MELVIO lineages, especially without diploid outgroups from sister sections (e.g., *Rubellium* and *Andinium*). Gene flow by introgression between species potentially occurs between closely related species only, i.e., within the same subsection, and can be ruled out for this dataset (e.g., Marcussen et al., 2015).

**Figure 5:**
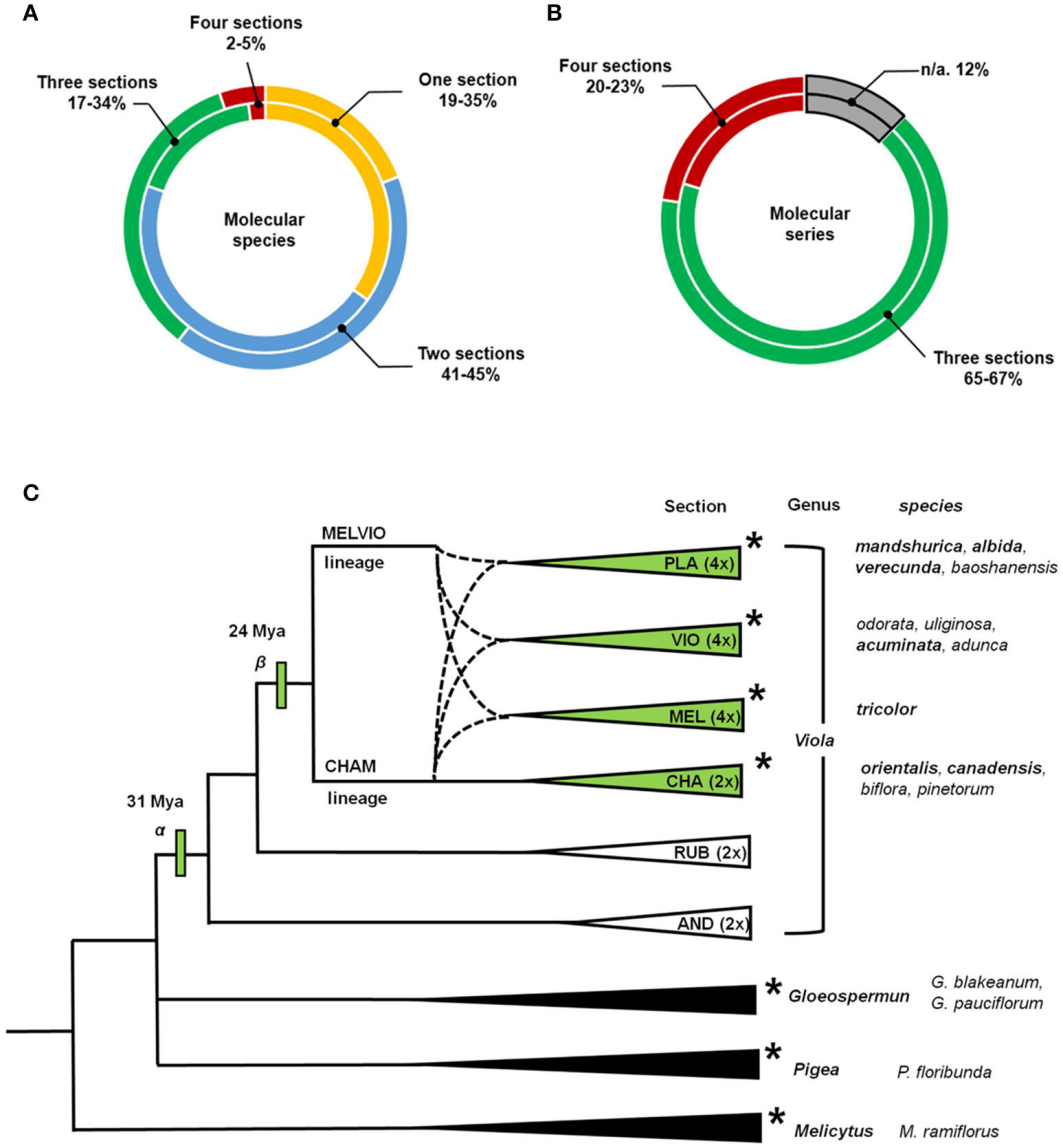
Distribution of molecular species and molecular series over the established phylogenetic relationships of *Viola*. Proportions of the total number of cyclotide molecular species and molecular series found in four different *Viola* sections. **(A)** The values shown in the outer rings shows results obtained by considering numbers of molecular species, and while those in the inner rings show results obtained based on numbers of precursor sequences. **(B)** The values shown in the outer rings shows results obtained by considering numbers of molecular series, and while those in the inner rings show results obtained based on numbers of precursor sequences. Those results are based on data for complete sequences. The result based on both complete and partial sequences is shown in Supplementary Figure 4. **(C)** Phylogenetic relationships between sections and genera of Violaceae used in the analyses conducted within this work. *Viola* Sections are abbreviated as follows: *Plagiostigma* as PLA, *Viola* as VIO, *Melanium* as MEL, *Chamaemelanium* as CHA, *Rubellium* as RUB, and *Andinium* as AND. Dotted lines indicate the complex ancestries of the three allotetraploid sections *Melanium, Plagiostigma*, and *Viola*, all derived from hybridization between the CHAM and MELVIO lineages 15–20 Ma ago. Genera and sections studied using transcriptomic methods are indicated with asterisks (^*^). The total number of species within *Viola* is estimated to be 580–620, most of which (61–65%) belong to these four sections. The first ancestor of the genus (α) is dated to 31 Mya, and the common ancestor of the four studied sections (β) is dated to 24 Mya (Marcussen et al., 2015). The phylogeny of *Viola* is based on the work of Marcussen et al. (2015) and that of Violaceae is based on the work of Wahlert et al. (2014).

The current analysis revealed several points about the distribution of cyclotide precursors within the genus *Viola*. Firstly, the classification into infrageneric sections is reflected in the distribution of the cyclotide precursors. Some molecular species occurred sporadically or commonly across the four infrageneric sections: 2% of molecular species were found across all sections, and 5% of the studied precursor sequences belong to those molecular species (Figure 5; see Supplementary Figure 4). Also, 17% of molecular species (34% of precursor sequences) were found across three sections, and 45% of molecular species (41% of precursor sequences) were found in at least two sections. This indicates that the molecular species likely originated both from the common ancestor of *Viola* sections and from the hybridization between the parental lineages. Also, there are some molecular species that could have occurred by the genetic changes from *Viola* speciation. It should be emphasized that the degree of concurrence is likely higher at the genomic level than at the transcriptomic level estimated from the current study, because all genomic presence of the molecular species can not be captured by the transcriptome. Some molecular species might not be expressed, and the RNA sequence of some molecular species might be degraded during the transcriptomic assay.

Secondly, signature sequences are conserved between *Viola* and other Violaceae genera, i.e., *Gloeospermum, Melicytus*, and *Pigea* (Supplementary Figure 5). In particular, the sequence signature of the NTR [−9, −8] region is largely conserved in the Möbius and bracelet lineages: Y^−9^-A^−8^ is found in the Möbius lineages of *Viola* and *Melicytus*, G^−9^-A^−8^ in the bracelet lineages of *Viola* and *Gloeospermum*, and H^−9^-S^−8^ in the bracelet lineages of *Viola* and *Pigea*. In addition, the sequence signature of the NTPP [−56, −38] is conserved in the bracelet lineage. That region shows high sequence similarity between some precursors, i.e., molecular species NS2 and HS3 from the genus *Viola* and precursors from *Melicytus* and *Pigea*, respectively. Interestingly, the protein sequence of Gpc3, a precursor belonging to the bracelet lineage, is identical in both *Viola* and *Gloeospermum*. These observations indicate that these sequence signatures have been conserved at least since the divergence of their most recent common ancestor some 50 million years ago (Marcussen, Wahlert et al., in prep.).

Thirdly, the cyclotide sequence diversity of *Viola* is expected to be dependent on the differentiation into sections. It is estimated that 67–88% of the *Viola* sections are allopolyploids (Marcussen et al., 2015) that combine genomes from different diploid lineages. Also, increase in ploidy has been shown experimentally to increase also sequence diversity of cyclotides by mutation, e.g., in *Oldenlandia affinis* (Seydel et al., 2007). However, the cyclotide sequence diversity could not only be very huge by the sequence variations within molecular species, but also be limited by sharing the same molecular species across different sections. The sequence diversity can be large by allowing conservative substitution within the same molecular species. Under the current classification (Marcussen et al., 2015), *Viola* comprises at least 16 sections and some 600 species, of which 10 sections (~454 species, 76%) possess at least one CHAM genome, either alone or, as a result of allopolyploidization, in combination with other CHAM genomes or MELVIO genomes. These 10 sections include the four (~380 species, 63%) inspected by the current study; and the current depth analysis reveals that most of cyclotide precursor sequences across different sections are likely grouped into the molecular species. The precursors identified in the current transcriptome and those reported previously all exhibit striking sequence similarity (Zhang et al., 2009, 2015b; Hellinger et al., 2015; Slazak et al., 2015).

### Expression profiles of cyclotides and their structural diversity

The expression profiles of cyclotides were analyzed at transcriptomic and peptidomic levels both. At the transcriptomic level, expression levels of precursor RNA were evaluated by their FPKM values. The expression of those sequences cyclotide sequences were then assayed at the peptidomic level by LC-MS.

At the transcriptomic level, a structurally diverse set of cyclotides were found, including archetypical, linear, hybrid and the novel types of cyclotides described above. In the bracelet lineage, the majority of cyclotide precursors were archetypical bracelets (73%, 125/171). In the Möbius lineage, the proportion of archetypical Möbius (33%, 26/78) was only second largest after the structural hybrids (35%, 28/78). However, judged by FPKM values, the expression levels of archetypical cyclotides were highest within each lineage. Most archetypical precursors have FPKM values higher than 150, i.e., 64% (= 9/14) and 79% (= 43/54) of the precursors in Möbius lineage and in bracelet lineage, respectively have values larger than the cutoff at 150 (Supplementary Table 4). On the other hand, precursors of novel cyclotides have low FKPM values, i.e., 53% (= 7/13) and 100% (= 13/13) of the precursors of novel cyclotides in the Möbius lineage and in the bracelet lineage, respectively. Moreover, in the Möbius lineage, 50% (= 6/12) of the precursors encoding structural hybrids exceed the FPKM-cutoff value.

**Figure 6:**
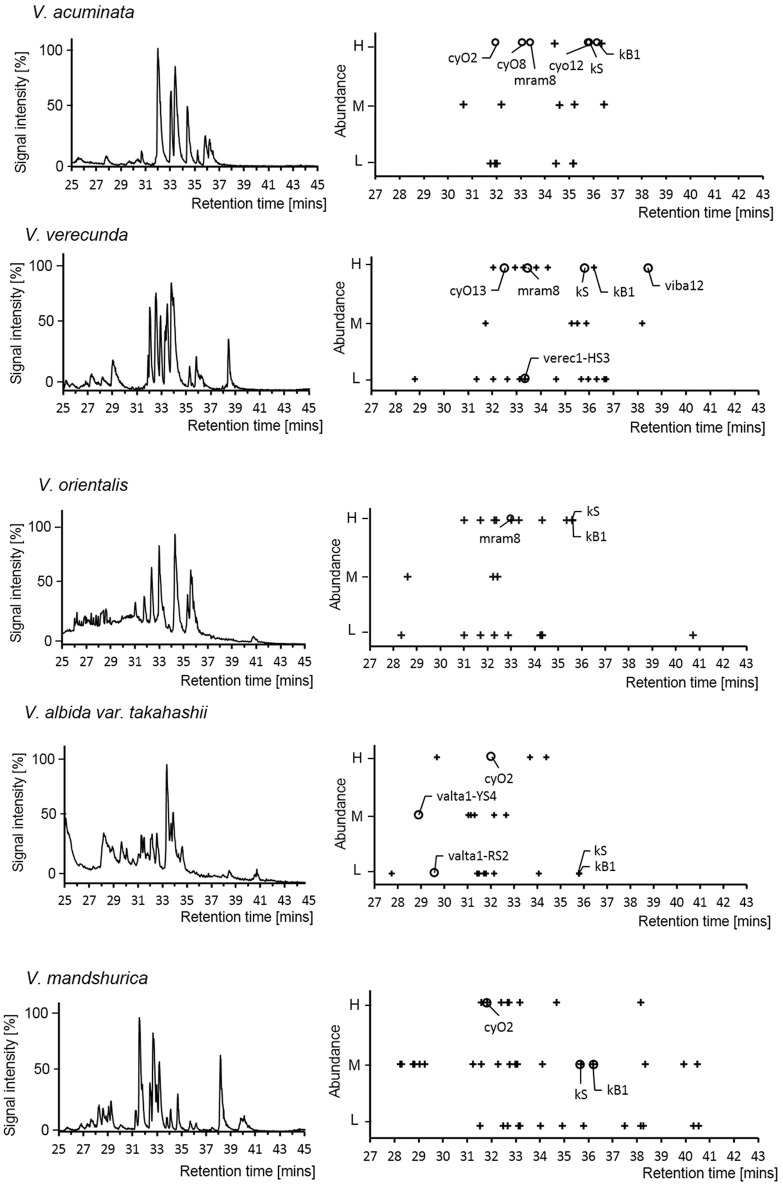
Cyclotide expression on peptidomic level. LC-MS chromatogram of the five *Viola* species used for transcriptome assays in current study (left panel). The abundance of cyclotides is linked to their signal intensity (Right panel). For each *Viola* species, the LC-MS chromatogram (Base Peak Ion) and mass distribution of the cyclotides are shown with retention times and abundance levels (*H*igh/*M*edium/*L*ow). The symbols + and ◦ denote cyclotides that were not found in the transcriptome and were found in the transcriptome, respectively. The cyclotides (kB1, kS, cyO2, cyO8, and cyO12) were confirmed by comparing the retention time and isotopic mass derived from the LC-MS of *V. tricolor* and *V. odorata* as reference chromatogram.

At the peptidomic level, numbers ranging from 19 to 44 cyclotides were detected in each of the *Viola* species. This number is similar to the number of precursor sequences found at transcriptomic level (23 to 37). Only few of the cyclotides (4–26%) were detected at both levels (Figure 6; see Supplementary Table 5). Most of those cyclotides were archetypical Möbius (kB1 and kS) and archetypical bracelets (cyO2, cyO8, cyO13, mram8, and viba12). Only two of the novel cyclotides were found at both levels, and both were found eluting with very early retention time and at low protein levels of abundance. Cyclotides found at the peptidomic-level only were identified by matching their calculated MW, assuming that they could belong to any type, e.g., be hybrid, archetypical Möbius and bracelet. Discrepancy between expression at transcriptomic- and peptidomic levels has been reported previously (Burman et al., 2010; Hellinger et al., 2015). In those studies cyclotides expressed as peptides were all hybrids, or archetypical Möbius or bracelets. The failure of detection the novel cyclotides using LC-MS could be either from the large hydrophilicity of those novel cyclotides (Supplementary Figure 6) or from their low expression at the peptidomic level. Interestingly, kS (varv A) and kB1 were not found in the transcriptome from two *Viola* species (*V. orientalis* and *V. albida* var. *takahashii*), whereas these particular cyclotides were found at the protein level in all five *Viola* species. Such expression differences could be related to the molecular stability and regulation mechanism. However, it is clear that cyclotides accumulate after their expression in plant tissues.

### Major sequence changes are linked to neofunctionality

**Figure 7:**
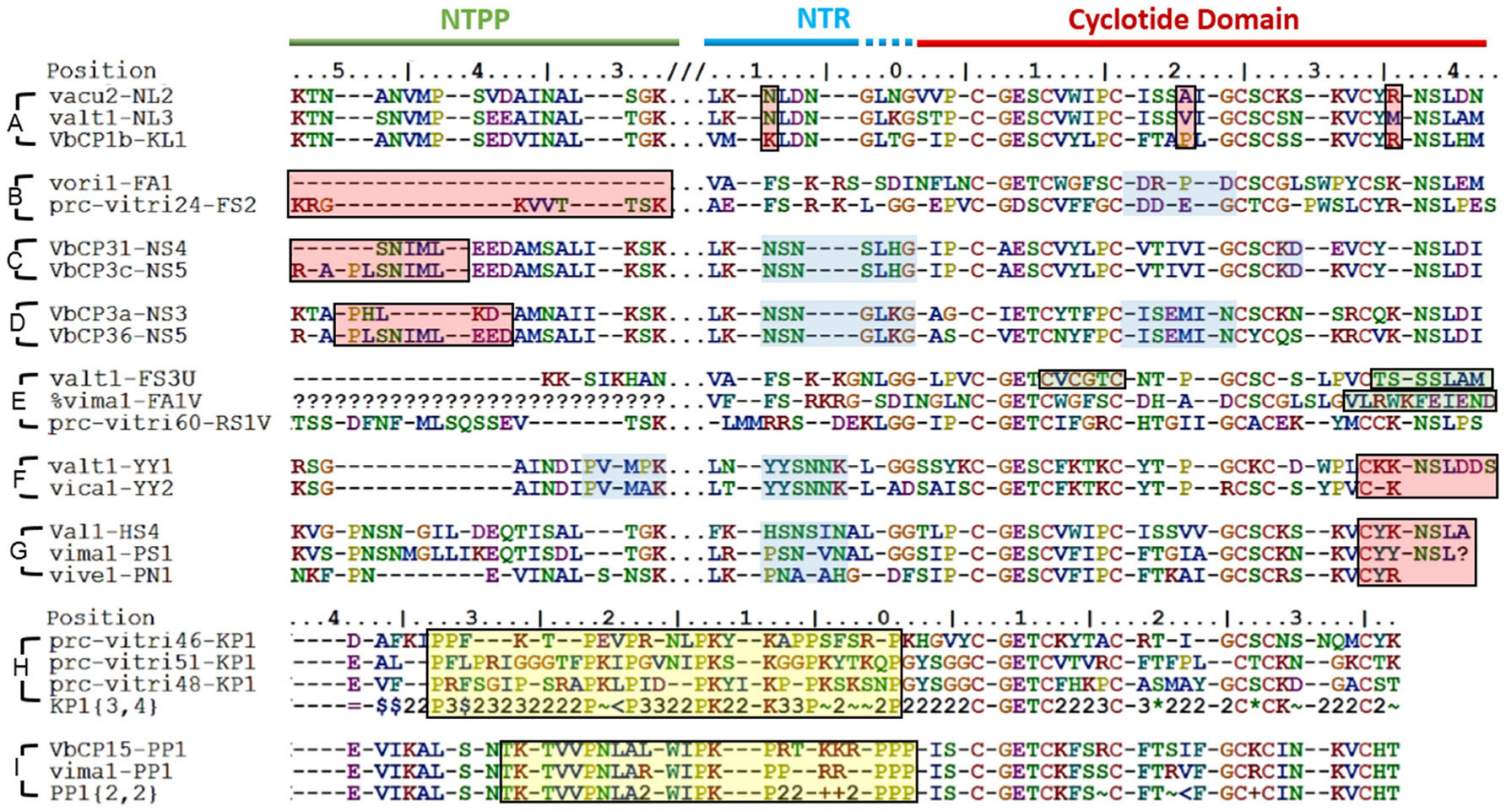
Sequence diversity between closely related molecular species. Some molecular species differ by only a few residues, e.g., **(A)** while others exhibit pronounced sequence variation, e.g., **(B–D)** Sequence differences are highlighted by red boxes. Common sequence traits (indicated by blue boxes) are found in closely related precursors. **(E)** Differences in the residues forming the CCK structural motif (green boxes). Additional cysteines are found in loop 2 of valt1-FS3U and loop 6 of prc-vitri60-RS1V. The cyclisation-enabling N/D residues are absent in loop 6 of valt1-FS3U. Vima1-FA1V lacks a cysteine residue in loop 6, which is elongated. **(F)** and **(G)**. Emergence of a cyclic backbone in the Möbius and bracelet lineages, respectively. Both lineages exhibit large sequence variation in the C-terminal loop 6. **(H,I)** Proline-rich sequence regions (indicated by yellow boxes).

In some cases, the differences between the precursor sequences of closely related molecular species were small (Figure 7A) while in other they were quite large (Figures 7B–D). In this context, a large difference is defined as an indel of more than five residues. With the exception of these indel regions, the precursor sequences are homologous, and the homologous sequence regions contain unique sequence traits found only in those molecular species. This implies that despite their large sequence differences, those molecular species are closely related. Such an outcome is analogous to that predicted by the theory of punctuated equilibrium (Eldredge and Gould, 1997), which describes evolutionary trends and speciation at the organism level. Another possible explanation for the observations is that mutations gradually accumulate in cyclotide precursors, leading to the loss or inactivation of intermediate sequences.

Some of the cyclotide domains exhibited unusual sequences, which fell into three main groups (Figure 7E). The first such group contained sequences whose cyclotide domains have an uneven number of cysteines (five or seven). Having uneven number of cysteines makes the formation of a cystine knot impossible, and can create the possibility of forming disulfide bridges not associated with the cystine knot, as well as the potential for dimerization. The second group consists of sequences that lack a residue required for AEP-mediated cyclisation, i.e., an N or D residue in loop 6. Abnormal precursor sequences of this sort are presumably the result of mutations in the genes encoding cyclic cyclotide precursors rather than being inherited from an ancestral linear cyclotide because they exhibit strong sequence similarity with the former class of precursors in both loop 6 and the C-terminal tail. Conversely, typical linear cyclotide precursors have short loop 6 sequences, lack a C-terminal tail, and are quite abundant. The third group of abnormal precursor sequences included sequences with one or more unusually short or long loops. Only five such sequences were identified (among a total of 283), but the three types of abnormality often co-occurred in the same precursor. For example, valt1-FS3U contains one additional cysteine in loop 2, and lacks an N/D residue in loop 6. All of these precursors are only weakly expressed.

The cyclic backbone seems to have emerged by molecular speciation. Comparing sequences from linear and cyclic molecular species, i.e., the cyclic YY1 and linear YY2 in Möbius lineage, and the cyclic PS1 and HS4 to the linear PN1 in the bracelet lineage (Figures 7F,G), suggests that linearity is likely to be the primitive (ancestral) trait, rather than linear cyclotides having evolved from cyclic ancestors via the mutational loss of the N/D residue in loop 6 that is needed for cyclisation. Although the cyclotide precursor sequences are too variable at the nucleotide level to resolve the relationships between linear and bracelet (Huson and Bryant, 2006), linearity being ancestral is supported by three facts. First, linear cyclotides that have undergone such mutational losses normally have elongated loop 6 sequences with mutations in the C-terminal tail. Second, the prodomain sequences of these molecular species are well-defined (all of them share the same sequence signature). Third, the molecular species corresponding to these putative primitive linear cyclotides are found in all four studied sections of *Viola* and also in other genera (Mra13).

Some precursors sequences could also be grouped as rich in prolines (Figures 7H,I). The common structure-based criterion used to identify mature Möbius cyclotides is the presence of a *cis* Pro in loop 5. Classification based on this approach is not generally consistent with the evolutionary classification into lineages: many precursor sequences assigned to the Möbius lineage lack this Pro residue in loop 5 (P32) of the cyclotide domain. Examples include the molecular species YA1, HF1, and YS1-3, which exhibit appreciable sequence similarity with archetypical Möbius cyclotides in loops 2 and 3. These sequence intermediates, which have characteristics of both bracelet and Möbius cyclotides, have been classified as the hybrid subfamily, and it has been suggested that they are genetic chimeras of bracelets and Möbius cyclotides (Daly et al., 2006). Our analysis suggests that this is not the case, at least in the Violaceae. Instead, these structural hybrids and archetypical Möbius cyclotides seem to originate from the molecular species YY1 of the Möbius lineage, which in turn is closely related to the linear ancestor YY2.

**Figure 8:**
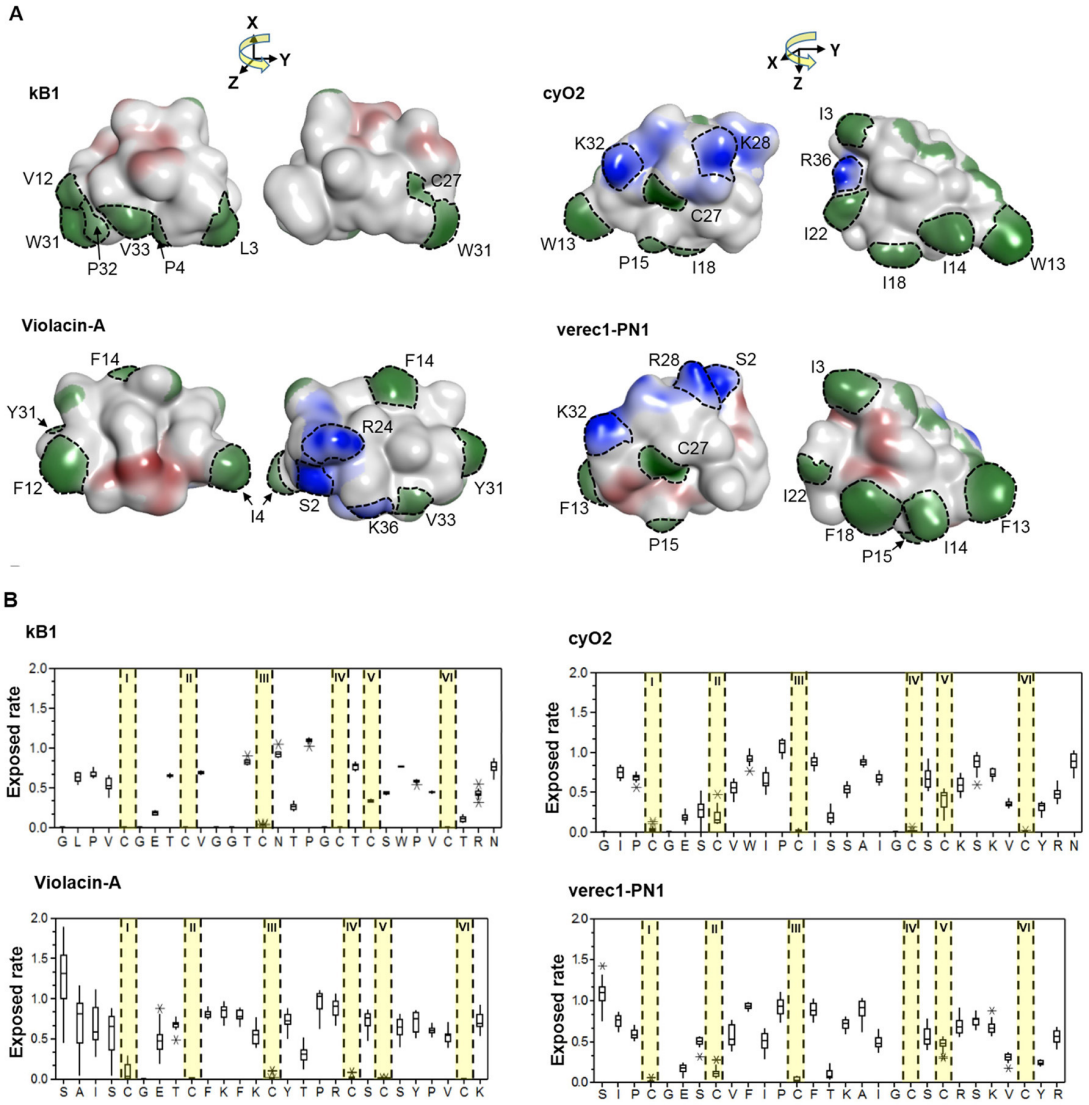
Membrane-interactive properties of linear and cyclic cyclotides within the Möbius and bracelet lineages. **(A)** Structures of kalata B1 and violacin A from the Möbius lineage, and cyO2 and verec1-PN1 from the bracelet lineage. Surface-exposed side-chains are shown in blue if positively charged, red if negatively charged, and green if hydrophobic. Notably, the N-terminal residues (S2) of the linear cyclotides always give rise to an exposed positive charge on the molecular surface. The numbering of residues follow the consensus sequence of the cyclotide domain in Figure 3. **(B)** Exposed ratios of linear and cyclic cyclotides' residues within each lineage. The exposure ratio compares the solvent-exposed surface area (SASA) of each residue in a protein to its SASA in the tripeptide Gly-X-Gly (ψ = ϕ = 180°); the more exposed the residue is, the greater the value. With the exception of the residues in loop 6, residues of cyclic and linear cyclotides have similar exposure ratios at corresponding positions. For each residue, the boxes show the range between the first and third quartiles, and the upper and lower error bars represent the minimum and maximum exposure ratios, respectively.

A key question is whether the emergence of the cyclic backbone changed the properties of the membrane-interacting surfaces of the linear cyclotide sequences. To answer this question, we analyzed the structural traits (i.e., the exposed ratio and physicochemical properties) of the residues that differ between linear and cyclic cyclotides within Möbius and bracelet lineages. The lineages were considered separately, so the cyclic Möbius cyclotide kalata B1 was compared to the linear cyclotide violacin A from the Möbius lineage. Similarly, the cyclic bracelet cyclotide cyO2 was compared to the linear verec1-PN1 from the bracelet lineage. In both cases, the linear cyclotide appeared to be ancestral to its cyclic counterpart. Linear and cyclic cyclotides from the same lineage exhibit substantial sequence similarity, with extensive conservation of residues' physicochemical properties at key positions. This is illustrated by the example of the residues in loop 6 (Figure 8B). In the linear cyclotides, loop 6 is conformationally flexible, and the charged groups of the termini are largely exposed on the molecular surface (Figures 8A,B). The exposed ratios and physicochemical properties of the corresponding residues in the cyclic cyclotides are very similar to those in their linear counterparts, implying that the structural framework favoring membrane interaction emerged before the cyclic backbone.

**Figure 9:**
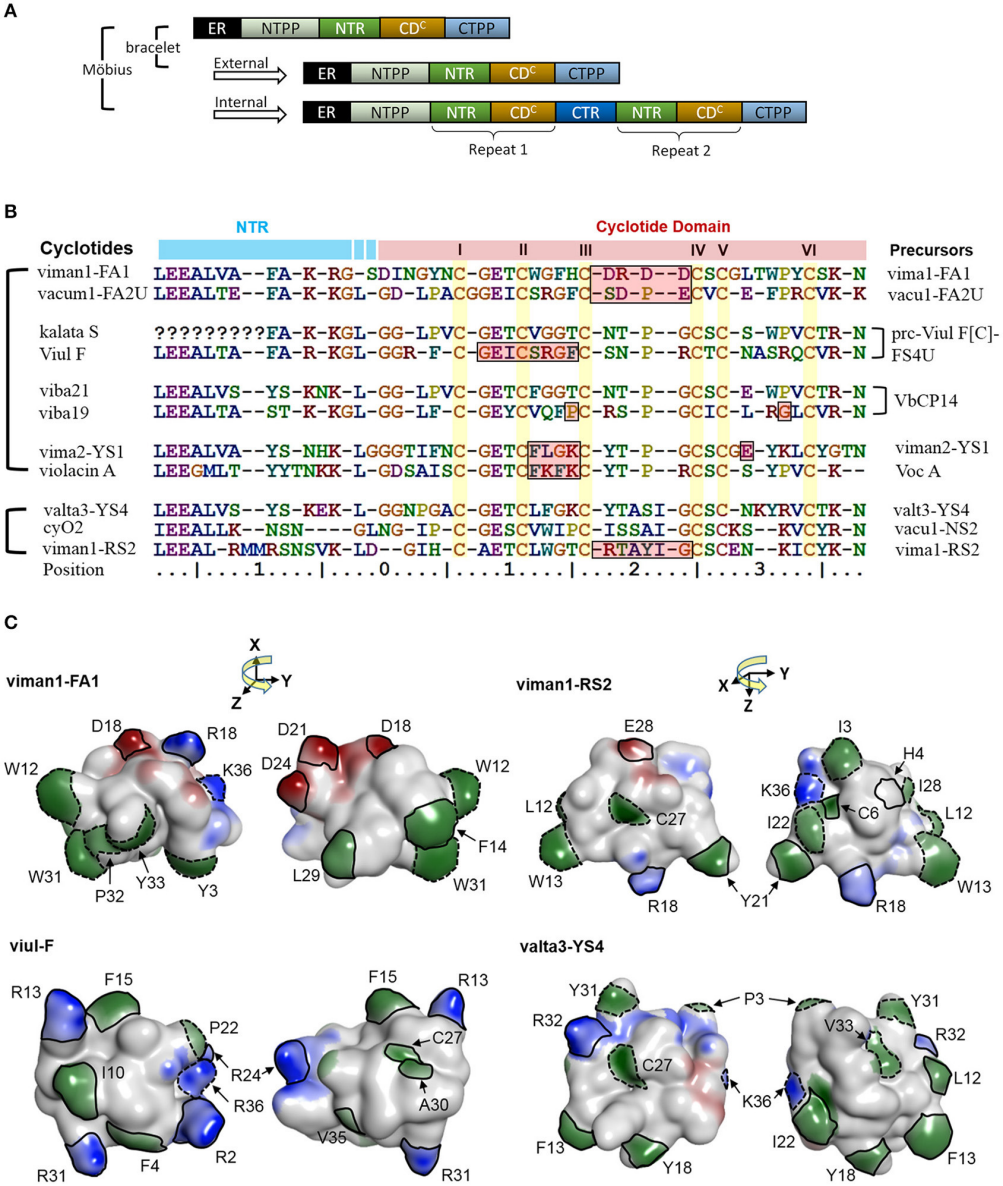
A potential link between precursor architecture and neofunctionality. **(A)** Suggested modes of duplication in precursor genes. In *Viola*, the precursor architecture differs between lineages. Precursors of bracelet lineages appear to contain only one modular domain (i.e., a concatenated NTR and a cyclotide domain), whereas precursors of the Möbius lineage may contain one to three such domains. The repeats in the Möbius lineage probably originated from internal gene duplication events. All precursors sharing the same architecture, in both the Möbius and bracelet lineages, probably originated from external gene duplication. **(B)** Sequence alignment between new types of cyclotides and previously identified archetypes. In the Möbius lineage, sequences vary between repeated cyclotide domains within the same precursor and cyclotide domains of different precursors. There are high levels of sequence conservation within repeated cyclotide domains, as exemplified by kB1 and kS within Vok1. However, the sequences of cyclotide domains in precursors containing multiple cyclotide domains in general are frequently very different, as exemplified by kalata S and viul F within prc-Viul F[C]-FS4U, and viba 19 and viba 21 within VbCP14. Of the precursor sequences shown in the figure, the molecular species YS7, which belongs to the Mobius lineage, has a similar sequence to archetypical bracelet cyclotides. For example, valta3-YS4 lacks a proline residue in loop 5 and has an elongated loop 3. Conversely, the molecular species FA1 contains lipophilic residues in loops 2 and 5, while loop 3 consists mainly of negatively charged residues. **(C)** Comparison of cyclotides' surfaces and electrostatic potentials. New types of cyclotides— viman-FA1, viman1-RS2, and valta3-YS4, and viul-F—are shown with their electrostatic potential surfaces. The distributions of their electrostatic potentials are generally dissimilar to those seen in typical bracelet and Mobius cyclotides. Residues with high exposed ratios are indicated by solid and dashed lines on the molecular surfaces. Residues highlighted with solid lines have dissimilar physicochemical properties to those found in the corresponding positions of archetypical cyclotides. Residues whose physicochemical properties match those of the archetypical cyclotides are indicated by dotted lines. Residues are numbered in accordance with their sequence positions in **(B)**. Negatively charged surface regions are colored in red, positively charged regions in blue, and hydrophobic regions in green. The numbering of residues follow the consensus sequence of the cyclotide domain in Figure 3.

Some of the cyclotide domains exhibit very high levels of sequence diversity between different molecular species. Some molecular species (e.g., YS1-4, RS2 and FA1) contain cyclotide domain sequences that are highly dissimilar to archetypical cyclotide sequences (Figure 9). We found that these molecular species occur across *Viola* sections, which means that they must have evolved before the differentiation of these sections some 15 million years ago. Although the biological function of all cyclotide forms is currently unclear, their maintenance within the genome suggests that they fulfill vital and divergent functions in their host plants.

It is likely that some cyclotides may have biological functions based on mechanisms other than membrane binding and disruption (Burman et al., 2011). For example, the cyclotides-FA1 might have different orientations to archetypical cyclotides, even when one compares them to others from the same lineage. They have lipophilic residues in loops 2 and 5 with large exposed areas on the molecular surface. The lipophilicity of this loop differs from that of archetypical cyclotides: the archetypal Möbius cyclotides do not have lipophilic residues in loop 2, and the archetypal bracelets do not have lipophilic residues in loop 5. In addition, loop 3 of the FA1 cyclotides is rich in negatively charged residues, making them unique among the molecular species found in the genus *Viola* and suggesting that their functions may be unrelated to membrane binding.

Contradicting previous hypotheses, it appears that the cyclotides of the Möbius lineage exhibit a somewhat greater diversity of sequence traits than those of the bracelet lineage. Such large sequence diversity of the Möbius lineage may be correlated with the combined occurrence of internal and external gene duplications in the cyclotide domain. Precursor architecture with such duplication of the cyclotide domain is illustrated in the Figure 9A. A large proportion of the precursors (72%; 56/78) belonging to the Möbius lineage contain multiple cyclotide domains that appear to have originated from internal duplication events. Conversely, no bracelet precursors having repeated cyclotide domains have yet been identified in the Violaceae. There are large sequence differences in certain precursors' cyclotide domains at positions close to the N-terminal prodomains, as can be seen by comparing the sequences of the FA1 and YS4 molecular species to the archetypal Möbius cyclotides varv A and kalata S (Figures 9B,C). Pronounced sequence differences also exist between repeated cyclotide domains in some individual precursors, as in the case of the precursor of Viul F. Together with the rest of our findings, this implies that combined internal and external duplications can synergistically produce large changes in cyclotides' sequences and structures, giving rise to new biological functions: neofunctionality.

## Author contributions

SP, TM, KJR, ID, AB, and UG designed experiments. SP carried out interpretation of precursor sequences, modeling and mass spectrometry. UG supervised the experiments. K-OY collected plant material for the transcriptome sequencing. EJ identified cyclotide genes from transcriptomic data. TM and SP performed the phylogenetic analyses. All authors contributed to the writing and revision of the manuscript.

### Conflict of interest statement

The authors declare that the research was conducted in the absence of any commercial or financial relationships that could be construed as a potential conflict of interest.
