# Supplementary material for: Cyclotide Evolution: Insights from the Analyses of Their Precursor Sequences, Structures and Distribution in Violets (Viola)
Source: Front Plant Sci. 2017 Dec 18;8:2058. doi: 10.3389/fpls.2017.02058 (PMC5741643; doi:10.3389/fpls.2017.02058)
Supplement: Supplementary file 1 [file Table1.DOCX]

**Supplementary Table 1.** Summary of *de novo* assembled transcriptomes for *Viola* species used in current study.

Summary of transcriptome for *V. verecunda*

| Info | All transcript contig |
| --- | --- |
| Total trinity transcript | 43,829 |
| Percent GC | 43.54 |
| Contig N50 | 1,117 |
| Maximum contig length | 6,585 |
| Minimum contig length | 201 |
| Median contig length | 450 |
| Average contig length | 710.27 |
| Total assembled bases | 31,130,250 |

Summary of transcriptome for *V. mandshurica*

| Info | All transcript contig |
| --- | --- |
| Total trinity transcript | 56,962 |
| Percent GC | 43.32 |
| Contig N50 | 1,211 |
| Maximum contig length | 7,771 |
| Minimum contig length | 201 |
| Median contig length | 472 |
| Average contig length | 760.9 |
| Total assembled bases | 43,342,248 |

Summary of transcriptome for *V. acuminata*

| Info | All transcript contig |
| --- | --- |
| Total trinity transcript | 51,846 |
| Percent GC | 43.47 |
| Contig N50 | 1,184 |
| Maximum contig length | 6,598 |
| Minimum contig length | 201 |
| Median contig length | 448 |
| Average contig length | 732.91 |
| Total assembled bases | 37,998,553 |

**Cont.**

Summary of transcriptome for *Viola albida* var. *takahashii*

| Info | All transcript contig |
| --- | --- |
| Total trinity transcript | 72,313 |
| Percent GC | 42.11 |
| Contig N50 | 1,189 |
| Maximum contig length | 9,956 |
| Minimum contig length | 201 |
| Median contig length | 440 |
| Average contig length | 733.81 |
| Total assembled bases | 53,064,295 |

Summary of transcriptome for *V. orientalis*

| Info | All transcript contig |
| --- | --- |
| Total trinity transcript | 54,589 |
| Percent GC | 44.5 |
| Contig N50 | 966 |
| Maximum contig length | 5,827 |
| Minimum contig length | 201 |
| Median contig length | 379 |
| Average contig length | 625.53 |
| Total assembled bases | 34,147,272 |
