# Supplementary material for: Cyclotide Evolution: Insights from the Analyses of Their Precursor Sequences, Structures and Distribution in Violets (Viola)
Source: Front Plant Sci. 2017 Dec 18;8:2058. doi: 10.3389/fpls.2017.02058 (PMC5741643; doi:10.3389/fpls.2017.02058)
Supplement: Supplementary file 2 [file Table2.DOCX]

**Supplementary Table 2.** Nomenclature of cyclotides and cyclotide precursors*.*

| Precursor name | Cyclotide name | Species |
| --- | --- | --- |
| vima  valt  vive  VbCP | viman  valta  viver  Viba | *Viola mandshurica* W.Becker  *Viola albida* Palib. var. *takahashii* (Nakai) Kitag.  *Viola verecunda* A.Gray  *Viola baoshanensis* W.S.Shu, W.Liu & C.Y.Lan |
| voc  prc-Viul  vacu  Vaf, Val | vodo  Viul  vacum  N.A. | *Viola odorata* L.  *Viola uliginosa* Bess.  *Viola acuminata* Ledeb.  *Viola adunca* Sm. |
| prc-vitri, prc-tricyclon | vitri, tricyclon | *Viola tricolor* L. |
| vori  Vbc  vica  prc-Vpf, prc-Vpl | vorie  vibi  vican  Vpf, Vpl | *Viola orientalis* W.Becker  *Viola biflora* L.  *Viola canadensis* L.  *Viola pinetorum* Greene |
