# Supplementary material for: Cyclotide Evolution: Insights from the Analyses of Their Precursor Sequences, Structures and Distribution in Violets (Viola)
Source: Front Plant Sci. 2017 Dec 18;8:2058. doi: 10.3389/fpls.2017.02058 (PMC5741643; doi:10.3389/fpls.2017.02058)
Supplement: Supplementary file 5 [file Table5.DOCX]

**Supplementary Table 5.**

From each *Viola* species, 19-44 cyclotides were detected in the protein level, and 23-37 cyclotide precursors were found in the transcriptomic level. Only 4-26% of cyclotides in protein-level were found in the transcriptomic-level. The 26% (5/19) of cyclotides were only found in both levels from *V. acuminata*, 17% (5/28) of cyclotides from *V. verecunda*, 4% (1/24) of cyclotides from *V. orientalis*, 14% (3/21) of cyclotides from *V. albida* var. *takahashii*, and 7% (3/44) of cyclotides from *V. mandshurica*. The cyclotides detected from both transcriptomic- and peptidomic levels are marked with ‘○’, and the cyclotides detected from peptidomic level only are marked with ‘+’. Even though some of the cyclotides (*e.g.* kS and kB1 in *V. orientalis*) are found from peptidomic level only, we assigned the sequence identities to those cyclotide entities based on their mass and retention times.

Fingerprint of *V. acuminata*

| Ret. time | Monoisotopic mass | Sig. Intensity | obs. | cal. | Δ mass | cyclotides |
| --- | --- | --- | --- | --- | --- | --- |
| 30.69 | 1171.00 ^3+^, 1755.53 ^2+^ | M | 3510.00 | - | - | + |
| 31.77 | 1455.12 ^2+^ | W | 2908.24 | - | - | + |
| 31.96 | 1101.17 ^3+^ | W | 3300.51 | - | - | + |
| 32.00 | 1448.17 ^3+^ | W | 2894.34 | - | - | + |
| 32.02 | 1047.10 ^3+^ , 1570.17 ^2+^ | S | 3138.30 | 3138.41 | 0.11 | cyO2 (○) |
| 32.22 | 1056.49 ^3+^, 1584.11 ^2+^ | M | 3166.47 | - | - | + |
| 33.10 | 1076.13 ^3+^, 1613.73 ^2+^ | S | 3225.39 | 3225.46 | 0.07 | cyO8 (○) |
| 33.41 | 1038.78 ^3+^, 1557.70 ^2+^ | S | 3113.34 | 3113.41 | 0.07 | mram8 (○) |
| 34.40 | 1071.84 ^3+^, 1607.13 ^2+^ | S | 3212.52 | - | - | + |
| 34.46 | 1532.10 ^2+^ | W | 3062.20 | - | - | + |
| 34.60 | 1030.08 ^3+^, 1544.64 ^2+^ | M | 3087.24 | - | - | + |
| 35.19 | 1009.75 ^3+^ | W | 3026.25 | - | - | + |
| 35.19 | 1015.77 ^3+^, 1523.15 ^2+^ | W | 3044.31 | - | - | + |
| 35.24 | 1032.77 ^3+^, 1548.69 ^2+^ | M | 3095.31 | - | - | + |
| 35.85 | 1446.13 ^2+^ | S | 2890.26 | 2890.18 | 0.08 | cyO12 (○) |
| 35.89 | 1439.07 ^2+^ | S | 2876.14 | 2876.16 | 0.02 | kS(○) |
| 36.22 | 1446.11 ^2+^ | S | 2890.22 | 2890.20 | 0.02 | kB1(○) |
| 36.35 | 1073.80 ^3+^, 1610.18 ^2+^ | S | 3218.40 | - | - | + |
| 36.49 | 1025.12 ^3+^, 1537.05 ^2+^ | M | 3072.36 | - | - | + |

**Cont.**

Fingerprint of *V. verecunda*

| Ret. time | Monoisotopic mass | Sig. Intensity | Obs. | Cal. | Δ mass | Cyclotides |
| --- | --- | --- | --- | --- | --- | --- |
| 28.77 | 1028.14 ^3+^ | W | 3081.42 | - | - | + |
| 31.31 | 1061.49 ^3+^ | W | 3181.47 | - | - | + |
| 31.70 | 1100.18 ^3+^ | M | 3297.54 | - | - | + |
| 32.02 | 1448.08 ^2+^ | W | 2894.16 | - | - | + |
| 32.03 | 1056.14 ^3+^ | S | 3165.42 | - | - | + |
| 32.52 | 1041.79 ^3+^, 1561.64 ^2+^ | S | 3122.37 | 3122.41 | 0.04 | cyO13(○) |
| 32.58 | 1126.50 ^3+^ | W | 3376.5 | - | - | + |
| 32.91 | 1072.50 ^3+^ | S | 3214.5 | - | - | + |
| 33.12 | 1454.14 ^2+^ | W | 2906.28 | - | - | + |
| 33.26 | 1087.48, 1630.63 | S | 3259.44 | - | - | + |
| 33.34 | 1601.28 ^2+^ | W | 3200.56 | - | - | + |
| 33.34 | 1601.28 ^2+^ | W | 3200.56 | 3200.34 | 0.22 | verec-HS3(○) |
| 33.46 | 1038.79 ^3+^ | S | 3113.37 | 3113.41 | 0.04 | mram8 (○) |
| 33.79 | 1033.45 ^3+^ | S | 3097.35 | - | - | + |
| 34.28 | 1096.16 ^3+^ | S | 3285.48 | - | - | + |
| 34.63 | 1556.06 ^2+^ | W | 3110.12 | - | - | + |
| 35.27 | 1019.79 ^3+^ | M | 3056.37 | - | - | + |
| 35.49 | 1028.79 ^3+^, 1543.20 ^2+^ | M | 3083.37 | - | - | + |
| 35.64 | 1565.52 ^2+^ | W | 3129.04 | - | - | + |
| 35.82 | 1439.07 ^2+^ | S | 2876.14 | 2876.16 | 0.02 | kS (○) |
| 35.86 | 1033.13 ^3+^ | M | 3096.39 | - | - | + |
| 35.94 | 1166.87 ^3+^ | W | 3497.61 | - | - | + |
| 36.19 | 1446.11 ^2+^ | S | 2890.22 | 2890.20 | 0.02 | kB1 (+) |
| 36.29 | 1457.03 ^2+^ | W | 2912.06 | - | - | + |
| 36.60 | 1584.67 ^2+^ | W | 3167.34 | - | - | + |
| 36.71 | 1592.63 ^2+^ | W | 3183.26 | - | - | + |
| 38.16 | 1105.51 ^3+^ | M | 3313.53 | - | - | + |
| 38.43 | 1051.46 ^3+^ | S | 3151.38 | 3151.41 | 0.03 | viba12 (○) |

**Cont.**

Fingerprint of *V. orientalis*

| Ret. time | Monoisotopic mass | Sig. Intensity | Obs. | Cal. | Δ mass | Cyclotides |
| --- | --- | --- | --- | --- | --- | --- |
| 28.35 | 1108.79 ^3+^ | W | 3323.37 | - | - | + |
| 28.61 | 1168.82 ^3+^ | M | 3503.46 | - | - | + |
| 31.01 | 1103.49 ^3+^ | S | 3307.47 | - | - | + |
| 31.05 | 1048.12 ^3+^ | W | 3141.36 | - | - | + |
| 31.75 | 1455.15 ^2+^ | W | 2908.30 | - | - | + |
| 31.76 | 1056.14 ^3+^ | S | 3165.42 | - | - | + |
| 32.23 | 1447.12 ^2+^ | M | 2892.24 | - | - | + |
| 32.29 | 1044.52 ^3+^ | W | 3130.56 | - | - | + |
| 32.34 | 1061.84 ^3+^, 1592.2 ^2+^ | S | 3182.52 | - | - | + |
| 32.38 | 1123.19 ^3+^ | S | 3366.57 | - | - | + |
| 32.42 | 1454.14 ^2+^ | M | 2892.24 | - | - | + |
| 32.93 | 1092.84 ^3+^ | W | 3275.52 | - | - | + |
| 33.01 | 1038.79 ^3+^, 1557.67 ^2+^ | S | 3113.37 | 3113.41 | 0.04 | mram8 (○) |
| 33.01 | 1040.17 ^3+^ | S | 3117.51 | - | - | + |
| 33.38 | 1063.13 ^3+^ | S | 3186.39 | - | - | + |
| 34.26 | 1006.42 ^3+^, 1509.18 ^2+^ | W | 3016.26 | - | - | + |
| 34.32 | 1019.14 ^3+^ | W | 3054.42 | - | - | + |
| 34.32 | 1025.11 ^3+^, 1537.20 ^2+^ | S | 3072.33 | - | - | + |
| 34.32 | 1032.49 ^3+^ | W | 3094.47 | - | - | + |
| 35.35 | 1024.44 ^3+^, 1536.25 ^2+^ | S | 3070.32 | - | - | + |
| 35.61 | 1439.15 ^2+^ | S | 2876.30 | 2876.16 | 0.14 | kS (+) |
| 35.61 | 1446.16 ^2+^ | S | 2890.32 | 2890.20 | 0.12 | kB1 (+) |
| 35.63 | 1458.64 ^2+^ | S | 2915.28 | - | - | + |
| 40.78 | 1015.45 ^3+^ | W | 3043.35 | - | - | + |

**Cont.**

Fingerprint of *V. albida* var. *takahashii*

| Ret. time | Monoisotopic mass | Sig. Intensity | Obs. | Cal. | Δ mass | cyclotides |
| --- | --- | --- | --- | --- | --- | --- |
| 27.71 | 1132.41 ^3+^ | W | 3394.23 | - | - | + |
| 28.90 | 1142.14 ^3+^ | M | 3423.42 | 3423.40 | 0.02 | ‘valta1-YS4’(○)  or ‘valta2-YS4’ (○) |
| 29.59 | 1117.5 ^3+^ | W | 3349.50 | 3349.41 | 0.09 | valta1-RS2 (○) |
| 29.66 | 1152.15 ^3+^, 1727.62 ^2+^ | S | 3453.45 | - | - | + |
| 31.03 | 1200.17 ^3+^ | M | 3597.51 | - | - | + |
| 31.13 | 1166.15 ^3+^, 1748.51 ^2+^ | M | 3495.45 | - | - | + |
| 31.30 | 1146.46 ^3+^, 1719.51 ^2+^ | M | 3436.38 | - | - | + |
| 31.44 | 1426.13 ^3+^ | W | 2850.26 | - | - | + |
| 31.50 | 1152.84 ^3+^ | W | 3455.52 | - | - | + |
| 31.73 | 1455.13 ^3+^ | W | 2908.26 | - | - | + |
| 31.77 | 1448.1 ^2+^ | W | 2894.20 | - | - | + |
| 32.03 | 1047.12 ^3+^, 1570.09 ^2+^ | S | 3138.36 | 3138.41 | 0.05 | cyO2 (○) |
| 32.14 | 1194.85 ^3+^ | W | 3581.55 | - | - | + |
| 32.14 | 1673.66 ^2+^ | M | 3345.32 | - | - | + |
| 32.67 | 1188.85 ^3+^ | M | 3563.55 | - | - | + |
| 33.69 | 1079.15 ^3+^ | S | 3234.45 | - | - | + |
| 34.04 | 1033.49 ^3+^ | W | 3097.47 | - | - | + |
| 34.38 | 1096.14 ^3+^, 1643.52 ^2+^ | S | 3285.42 | - | - | + |
| 35.79 | 1417.04 ^2+^ | W | 2832.08 | - | - | + |
| 35.79 | 1439.11 ^2+^ | W | 2876.22 | 2876.16 | 0.06 | kS (+) |
| 35.79 | 1446.13 ^2+^ | W | 2890.26 | 2890.20 | 0.06 | kB1(+) |

**Cont.**

Fingerprint of *V. mandschurica*

| Ret. time | Monoisotopic mass | Sig. Intensity | Obs. | Cal. | Δ mass | cyclotides |
| --- | --- | --- | --- | --- | --- | --- |
| 28.25 | 1186.54 ^3+^ | M | 3556.61 | - | - | + |
| 28.29 | 1187.91 ^3+^ | M | 3560.73 | - | - | + |
| 28.76 | 1182.50 ^3+^ | M | 3544.49 | - | - | + |
| 28.82 | 1061.41 ^3+^, 1591.62 ^2+^ | M | 3181.22 | - | - | + |
| 29.05 | 1181.14 ^3+^ | M | 3540.43 | - | - | + |
| 29.25 | 1175.83 ^3+^ | M | 3524.48 | - | - | + |
| 31.24 | 1078.85 ^3+^ | M | 3233.54 | - | - | + |
| 31.53 | 1448.13 ^3+^ | W | 2894.25 | - | - | + |
| 31.57 | 1037.10 ^3+^ | S | 3108.29 | - | - | + |
| 31.57 | 1037.62 ^3+^ | M | 3109.86 | - | - | + |
| 31.79 | 1047.09 ^3+^ | S | 3138.26 | 3138.41 | 0.15 | cyO2 (○) |
| 31.81 | 1079.85 ^3+^, 1619.34 ^2+^ | S | 3236.55 | - | - | + |
| 32.28 | 1051.41 ^3+^ | M | 3151.23 | - | - | + |
| 32.41 | 1092.23 ^3+^, 1637.8 ^2+^ | S | 3273.70 | - | - | + |
| 32.49 | 1047.73 ^3+^ | W | 3140.20 | - | - | + |
| 32.67 | 1080.87 ^3+^ | S | 3239.62 | - | - | + |
| 32.67 | 1081.69 ^3+^ | W | 3242.06 | - | - | + |
| 32.72 | 1057.79 ^3+^, 1586.21 ^2+^ | S | 3170.36 | - | - | + |
| 32.76 | 1076.21 ^3+^ | M | 3225.63 | - | - | + |
| 32.98 | 1071.50 ^3+^ | M | 3211.49 | - | - | + |
| 33.06 | 1089.89 ^3+^ | M | 3266.66 | - | - | + |
| 33.13 | 1072.58 ^3+^ | W | 3214.73 | - | - | + |
| 33.17 | 1038.71 ^3+^ | S | 3113.14 | - | - | + |
| 33.17 | 1039.26 ^3+^ | W | 3114.77 | - | - | + |
| 34.02 | 1081.28 ^3+^ | W | 3240.84 | - | - | + |
| 34.09 | 1080.51 ^3+^ | M | 3238.53 | - | - | + |
| 34.68 | 1062.43 ^3+^, 1593.03 ^2+^ | S | 3184.29 | - | - | + |
| 34.93 | 1069.15 ^3+^ | W | 3204.46 | - | - | + |
| 35.63 | 1067.15 ^3+^ | M | 3198.46 | - | - | + |
| 35.68 | 1439.03 ^2+^ | M | 2876.06 | 2876.16 | 0.10 | kS (○) |
| 35.79 | 1423.95 ^2+^ | W | 2845.90 | - | - | + |
| 35.79 | 1431.06 ^2+^ | W | 2860.12 | - | - | + |
| 36.19 | 1174.21 ^3+^ | M | 3519.64 | - | - | + |
| 36.19 | 1446.14 ^2+^ | M | 2890.28 | 2890.20 | 0.08 | kB1(○) |
| 37.50 | 1034.90 ^3+^ | W | 3101.71 | - | - | + |
| 38.16 | 1023.27 ^3+^ | W | 3066.82 | - | - | + |
| 38.16 | 1029.00 ^3+^, 1543.01 ^2+^ | S | 3083.99 | - | - | + |
| 38.26 | 1005.24 ^3+^, 1507.28 ^2+^ | W | 3012.72 | - | - | + |
| 38.34 | 1052.11 ^3+^ | M | 3153.34 | - | - | + |
| 39.93 | 1019.60 ^2+^, 1528.76 ^3+^ | M | 3055.80 | - | - | + |
| 40.34 | 1033.00 ^3+^ | W | 3096.00 | - | - | + |
| 40.50 | 1041.34 ^3+^ | M | 3121.01 | - | - | + |
| 40.55 | 1040.31 ^3+^ | W | 3117.93 | - | - | + |
| 45.42 | 1014.49 ^3+^ | W | 3040.46 | - | - | + |
