# Supplementary material for: Cyclotide Evolution: Insights from the Analyses of Their Precursor Sequences, Structures and Distribution in Violets (Viola)
Source: Front Plant Sci. 2017 Dec 18;8:2058. doi: 10.3389/fpls.2017.02058 (PMC5741643; doi:10.3389/fpls.2017.02058)
Supplement: Supplementary file 10 [file Image1.PDF]

Supplementary Figure 1. The classification of cyclotides based on sequence signatures

Out of 283 precursor sequence from genus *Viola*, the 249 cyclotide precursors were classified into two lineages (*i.e.* Möbius and bracelet), and further 13 molecular series and 46 molecular species (*i.e.* 5 molecular series and 14 molecular species within Möbius lineage, and 8 molecular series and 32 molecular species within bracelet lineage). Those 249 entailed precursor sequences are shown with multiple sequence alignment in this figure, and the 34 unclassified precursor sequences are in the Figure S2.

A. Precursor sequences belong to Möbius lineage

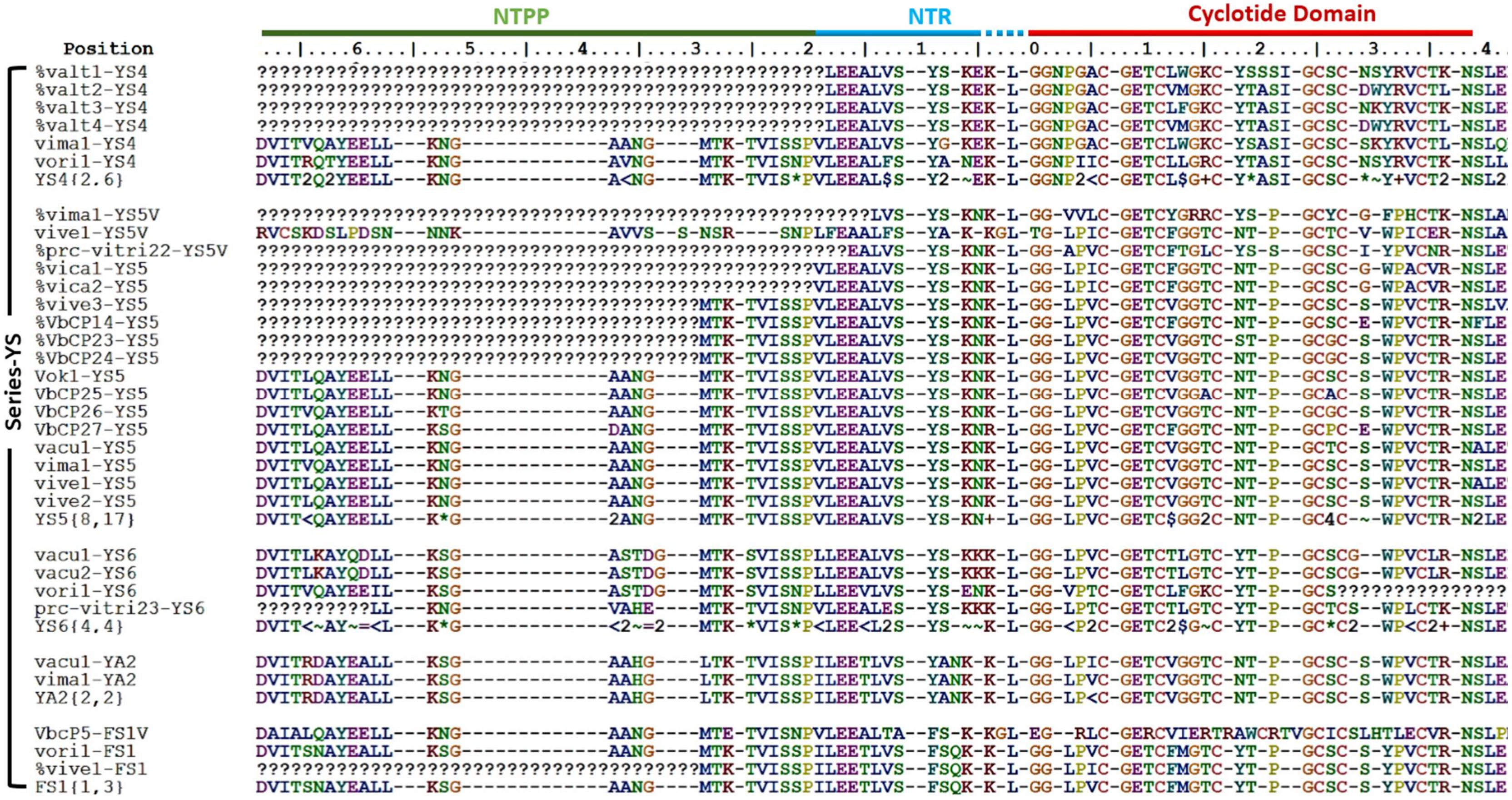

Cont.

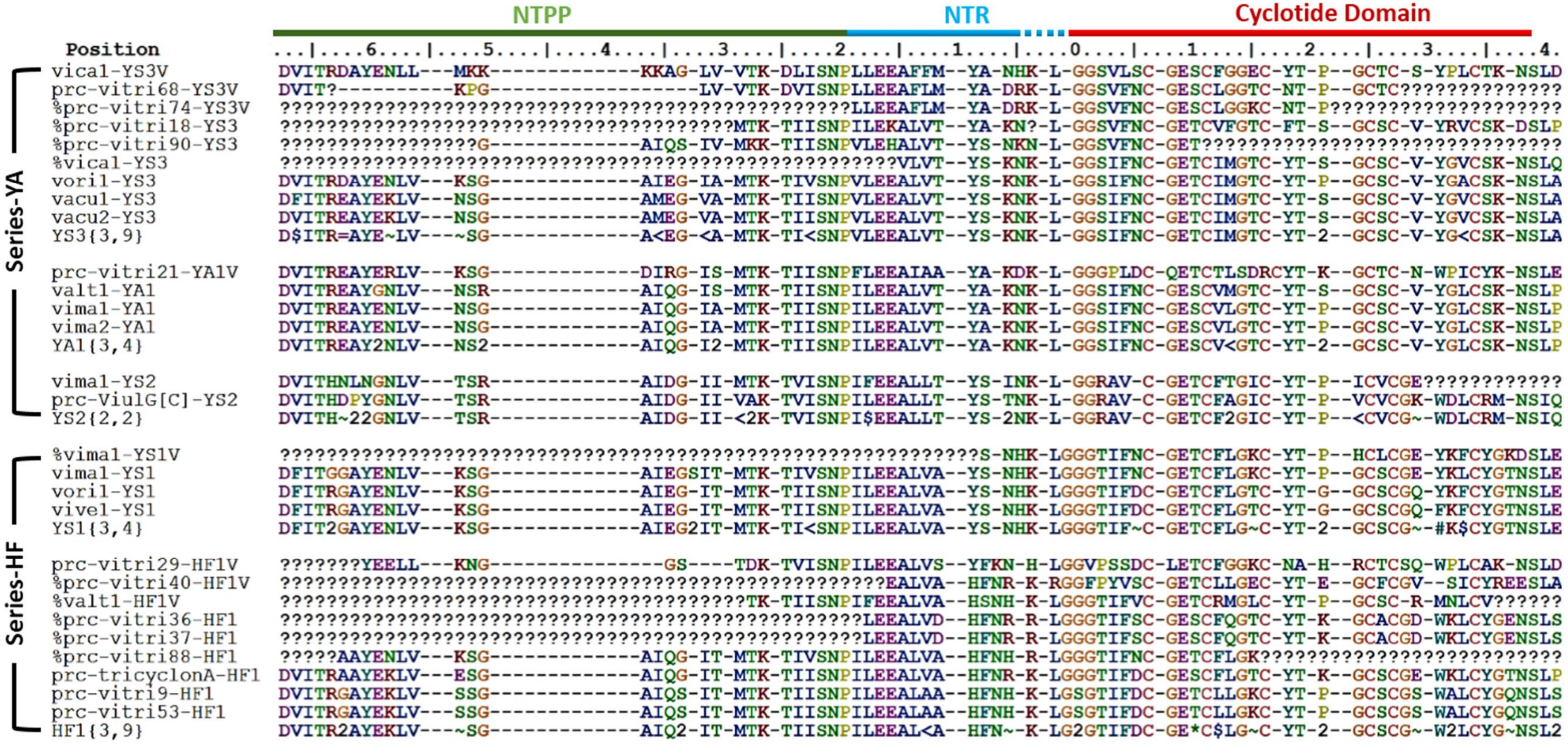
NTPP
NTR
Cyclotide Domain
Position ...|....6....|....5....|....4....|....3....|....2....|....1....|....0....|....1....|....2....|....3....|....4.
vica1-YS3V DVITRDAYENLL---MKK-------------KKAG-LV-VTK-DLISNPLLEEAFFM--YA-NHK-L-GGSVLSC-GESCFGGEC-YT-P--GCTC-S-YPLCTK-NSLD
prc-vitri68-YS3V DVIT?----------KPG------------------LV-VTK-DVISNPLLEEAFLM--YA-DRK-L-GGSVFNC-GESCLGGTC-NT-P--GCTC??????????????
%prc-vitri74-YS3V ???LLEEAFLM--YA-DRK-L-GGSVFNC-GESCLGGKC-NT-P????????????????????
%prc-vitri18-YS3 ??MTK-TIISNPILEKALVT--YA-KN?-L-GGSVFNC-GETCVFGTC-FT-S--GCSC-V-YRVCSK-DSLP
%prc-vitri90-YS3 ?????????????????G-------------AIQS-IV-MKK-TIISNPVLEHALVT--YS-NKN-L-GGSVFNC-GET????????????????????????????????
%vica1-YS3 ??VLVT--YS-KNK-L-GGSIFNC-GETCIMGTC-YT-S--GCSC-V-YGVCSK-NSLQ
vori1-YS3 DVITRDAYENLV---KSG-------------AIEG-IA-MTK-TIVSNPVLEEALVT--YS-KNK-L-GGSIFNC-GETCIMGTC-YT-P--GCSC-V-YGACSK-NSLA
vacu1-YS3 DFITREAYEKLV---NSG-------------AMEG-VA-MTK-TIISNPVLEEALVT--YS-KNK-L-GGSIFNC-GETCIMGTC-YT-S--GCSC-V-YGVCSK-NSLA
vacu2-YS3 DVITREAYEKLV---NSG-------------AMEG-VA-MTK-TIISNPVLEEALVT--YS-KNK-L-GGSIFNC-GETCIMGTC-YT-S--GCSC-V-YGVCSK-NSLA
YS3{3,9} D$ITR=AYE~LV---~SG-------------A<EG-<A-MTK-TI<SNPVLEEALVT--YS-KNK-L-GGSIFNC-GETCIMGTC-YT-2--GCSC-V-YG<CSK-NSLA
prc-vitri21-YA1V DVITREAYERLV---KSG-------------DIRG-IS-MTK-TIISNPFLEEAIAA--YA-KDK-L-GGGPLDC-QETCTLSDRCYT-K--GCTC-N-WPICYK-NSLE
valt1-YA1 DVITREAYGNLV---NSR-------------AIQG-IS-MTK-TIISNPILEEALVT--YA-KNK-L-GGSIFNC-GESCVMGTC-YT-S--GCSC-V-YGLCSK-NSLP
vima1-YA1 DVITREAYENLV---NSG-------------AIQG-IA-MTK-TIISNPILEEALVT--YA-KNK-L-GGSIFNC-GESCVLGTC-YT-P--GCSC-V-YGLCSK-NSLP
vima2-YA1 DVITREAYENLV---NSG-------------AIQG-IA-MTK-TIISNPILEEALVT--YA-KNK-L-GGSIFNC-GESCVLGTC-YT-P--GCSC-V-YGLCSK-NSLP
YA1{3,4} DVITREAY2NLV---NS2-------------AIQG-I2-MTK-TIISNPILEEALVT--YA-KNK-L-GGSIFNC-GESCV<GTC-YT-2--GCSC-V-YGLCSK-NSLP
vima1-YS2 DVITHNLNGNLV---TSR-------------AIDG-II-MTK-TVISNPIFEEALLT--YS-INK-L-GGRAV-C-GETCFTGIC-YT-P--ICVCGE????????????
prc-ViulG[C]-YS2 DVITHDPYGNLV---TSR-------------AIDG-II-VAK-TVISNPILEEALLT--YS-TNK-L-GGRAV-C-GETCFAGIC-YT-P--VCVCGK-WDLCRM-NSIQ
YS2{2,2} DVITH~22GNLV---TSR-------------AIDG-II-<2K-TVISNPI$EEALLT--YS-2NK-L-GGRAV-C-GETCF2GIC-YT-P--<CVCG~-WDLCRM-NSIQ
%vima1-YS1V ??S-NHK-LGGGTIFNC-GETCFLGKC-YT-P--HCLCGE-YKFCYGKDSLE
vima1-YS1 DFITGGAYENLV---KSG-------------AIEGSIT-MTK-TIVSNPILEEALVA--YS-NHK-LGGGTIFNC-GETCFLGKC-YT-P--GCSCGE-YKLCYGTNSLE
vori1-YS1 DFITRGAYENLV---KSG-------------AIEG-IT-MTK-TIISNPILEEALVA--YS-NHK-LGGGTIFDC-GETCFLGTC-YT-G--GCSCGQ-YKFCYGTNSLE
vive1-YS1 DFITRGAYENLV---KSG-------------AIEG-IT-MTK-TIISNPILEEALVA--YS-NHK-LGGGTIFDC-GETCFLGTC-YT-G--GCSCGQ-FKFCYGTNSLE
YS1{3,4} DFIT2GAYENLV---KSG-------------AIEG2IT-MTK-TI<SNPILEEALVA--YS-NHK-LGGGTIF~C-GETCFLG~C-YT-2--GCSCG~-#K$CYGTNSLE
prc-vitri29-HF1V ???????YEELL---KNG----------------GS----TDK-TVISNPILEEALVS--YFKN-H-LGGVPSSDC-LETCFGGKC-NA-H--RCTCSQ-WPLCAK-NSLD
%prc-vitri40-HF1V ???EALVA--HFNR-K-RGGFPYVSC-GETCLLGEC-YT-E--GCFCGV--SICYREESLA
%valt1-HF1V ???TK-TIISNPIFEEALVA--HSNH-K-LGGGTIFVC-GETCRMGLC-YT-P--GCSC-R-MNLCV??????
%prc-vitri36-HF1 ??LEEALVD--HFNR-R-LGGGTIFSC-GESCFQGTC-YT-K--GCACGD-WKLCYGENSLS
%prc-vitri37-HF1 ??LEEALVD--HFNR-R-LGGGTIFSC-GESCFQGTC-YT-K--GCACGD-WKLCYGENSLS
%prc-vitri88-HF1 ?????AAYENLV---KSG-------------AIQG-IT-MTK-TIVSNPILEEALVA--HFNH-R-LGGGTIFNC-GETCFLGK??????????????????????????
prc-tricyclonA-HF1 DVITRAAYEKLV---ESG-------------AIQG-IT-MTK-TIISNPILEEALVA--HFNR-K-LGGGTIFDC-GESCFLGTC-YT-K--GCSCGE-WKLCYGTNSLP
prc-vitri9-HF1 DVITRGAYEKLV---SSG-------------AIQS-IT-MTK-TIISNPILEEALAA--HFNH-K-LGSGTIFDC-GETCLLGKC-YT-P--GCSCGS-WALCYGQNSLS
prc-vitri53-HF1 DVITRGAYEKLV---SSG-------------AIQS-IT-MTK-TIISNPILEEALAA--HFNH-K-LGSGTIFDC-GETCLLGKC-YT-P--GCSCGS-WALCYGQNSLS
HF1{3,9} DVITR2AYEKLV---~SG-------------AIQ2-IT-MTK-TIISNPILEEAL<A--HFN~-K-LG2GTIFDC-GE*C$LG~C-YT-2--GCSCG~-W2LCYG~NSL2
Series-YA
Series-HF

Cont.

```
                                  NTPP                                         NTR              Cyclotide Domain
Position             ...|....6....|....5....|....4....|....3....|....2....|....1....|....0....|....1....|....2....|....3....|....4...

Series-YY
valt1-YY1            DFITRRAYDNLV---RSG-------------AINDIPV-MPK-TIISNPVLEEALLN--YYSNNK-L-GGSSYKC-GESCFKTKC-YT-P--GCKC-D-WPLCKK-NSLDDST
vima1-YY1            HFITRRAYDNLV---KSG-------------AINDDPV-MAK-TIISNPVLEDAPLT--YYSSNK-L-RGTELLC-GGFCRFSLC-RR----GCYC-D-FPRCMK-NSLDDST
YY1{2,2}             ~FITRRAYDNLV---+SG-------------AIND2PV-M2K-TIISNPVLE=A2LT--YYS*NK-L-2G*~$2C-G22C22*2C-22-2--GC2C-D-#P2C2K-NSLDDST

VocA-YY2             DFITRRAYDNLV---KSG-------------AIKDIPV-MAK-TIISNPVLEEGMLT--YYTNKK-L-GDSAISC-GETCFKFKC-YT-P--RCSC-S-YPVC-K
vica1-YY2            DFITRRAYDNLV---KSG-------------AINDIPV-MAK-TIISNPVLEEGLLT--YYSNNK-L-ADSAISC-GETCFKTKC-YT-P--RCSC-S-YPVC-K
YY2{2,2}             DFITRRAYDNLV---KSG-------------AI~DIPV-MAK-TIISNPVLEEG<LT--YY*N~K-L-2DSAISC-GETCFK2KC-YT-P--RCSC-S-YPVC-K

Series-FA
prc-vitri24-FS2      DFITRGAYEDLV---KRG-------------KVVT----TSK-TIIANPILEEAFAE--FS-R-K-L-GG-EPVC-GDSCVFFGC-DD-E--GCTCG-PWSLCYR-NSLPESY
prc-vitri28-FS2      DFITRGAYEDLV---KRG---------------------?SK-TIIANPILEEAFAE--FS-R-K-L-GG-EPVC-GDSCVFFGC-DD-E--GCTCG-PWSLCYR-NSLPESY
FS2{2.2}             DFITRGAYEDLV---KRG-------------2222----TSK-TIIANPILEEAFAE--FS-R-K-L-GG-EPVC-GDSCVFFGC-DD-E--GCTCG-PWSLCYR-NSLPESY

%vima1-FA1V          ?????????????????????????????????????????????????????RTVF--FS-RKRG-SDINGLNC-GETCWGFSC-DH-A--DCSCGLSLGVLRWKFEIENDG
vica1-FA1V           ---SRNSFDE---------------------------------NNS--LLLKEALVA--FA-K-RG-GDINGGLNCGETCWGFSC-DR-A--GCSCGWTTWPYCSDNSLDV
prc-vitri45-FA1V     ---LRNSF-E---------------------------------NNNDDHILEEALVL--FN-K----LD--GLNC-GETCWGFTC-NR-A--DCSCGFTYPYCSK-NSLDM
vica1-FA1            ---SRNSFDE---------------------------------NNS--LLLEEALVA--FA-K-RG-GDINGLNC-GETCWGFSC-DR-A--DCSC-WTWPYCSK-NS
vima1-FA1            ---SRNSFDEK--------------------------------NN---LILEEALVA--FA-K-RG-SDINGYNC-GETCWGFHC-DR-D--DCSCGLTWPYCSK-NSLDV
valt1-FA1            ---SRNSFDEK--------------------------------NS---LLLEEALVA--FA-K-RG-GDINGLNC-GETCWGFHC-DR-P--DCSCGLTWPYCAK-NSLDV
valt2-FA1            ---SRNSFDEK--------------------------------NS---LILEEALVA--FA-K-RG-SDINFLNC-GETCWGFSC-DR-H--DCSCGLSWPYCSK-NSLDE
vacu1-FA1            ---SRNSFDE---------------------------------NN---VLLEEALVA--FA-K-RG--DINGLNC-GETCWGFHC-DR-D--DCSCGLTWPYCSK-NSLHM
vori1-FA1            ---SRNSFDE---------------------------------NNS--LLLEEALVA--FS-K-RS-SDINFLNC-GETCWGFSC-DR-P--DCSCGLSWPYCSK-NSLEM
vacu2-FA1            ---SRNSF-E---------------------------------NNN--HLLEEALIA--FN-K-RG-TDLGGLNC-GETCWGFSC-DR-P--DCSCGLTWPYCSK-NSLDM
prc-vitri43-FA1      ---LRNSF-E---------------------------------NNNDDHILEEALVL--FN-K----LD--GLNC-GETCWGFSC-DR-D--DCSCGFTWPYCSK-NSLDM
prc-vitri44-FA1      ---LRNSF-E---------------------------------NNKNDHILEEALVL--FN-K----LD--GLNC-GETCWGFSC-DR-D--DCSCGFTWPYCSK-NSLDM
FA1{9,12}            ---2RNSF2E2--------------------------------N*2222<LEEAL<<--F2-K-23-4D232$NC-GETCWGF*C-DR-3--DCSC2$*WPYC2K-NSL~2
```

B. Precursor sequences belong to bracelet lineage

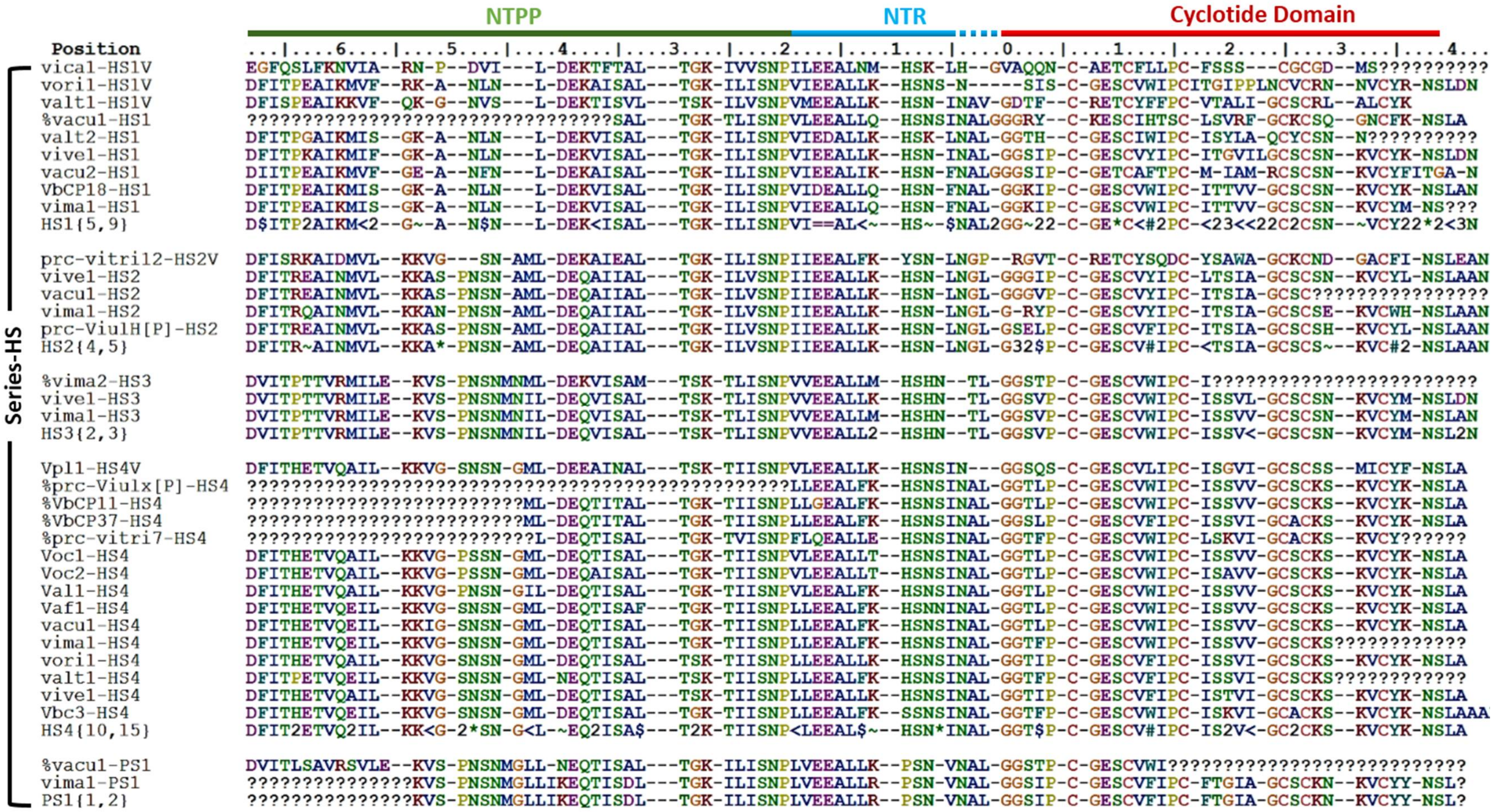

Cont.

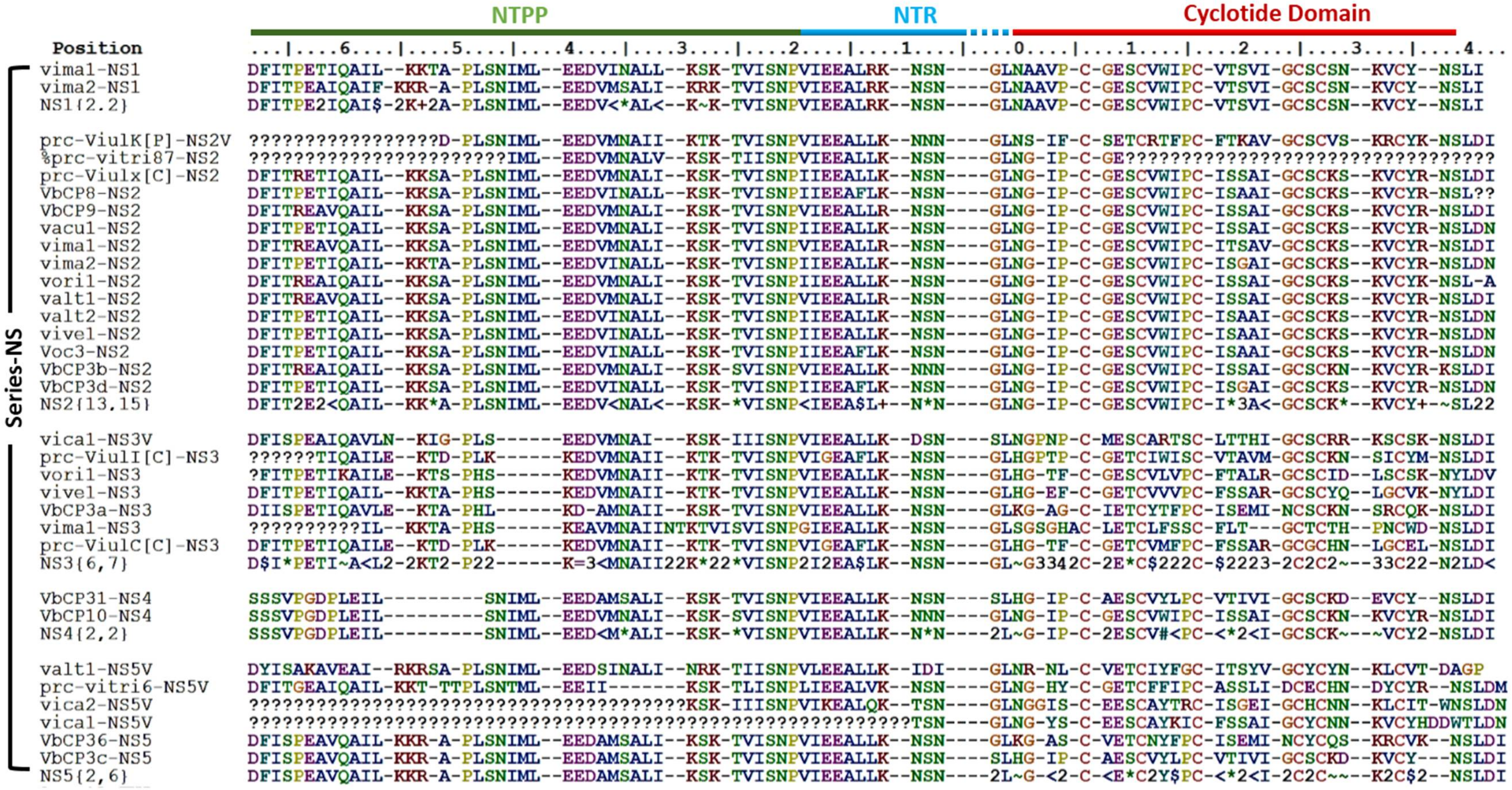
NTPP
NTR
Cyclotide Domain
Position
Series-NS
vima1-NS1
vima2-NS1
NS1{2,2}
prc-ViulK[P]-NS2V
%prc-vitri87-NS2
prc-Viulx[C]-NS2
VbCP8-NS2
VbCP9-NS2
vacu1-NS2
vima1-NS2
vima2-NS2
vori1-NS2
valt1-NS2
valt2-NS2
vive1-NS2
Voc3-NS2
VbCP3b-NS2
VbCP3d-NS2
NS2{13,15}
vica1-NS3V
prc-ViulI[C]-NS3
vori1-NS3
vive1-NS3
VbCP3a-NS3
vima1-NS3
prc-ViulC[C]-NS3
NS3{6,7}
VbCP31-NS4
VbCP10-NS4
NS4{2,2}
valt1-NS5V
prc-vitri6-NS5V
vica2-NS5V
vica1-NS5V
VbCP36-NS5
VbCP3c-NS5
NS5{2,6}

Cont.

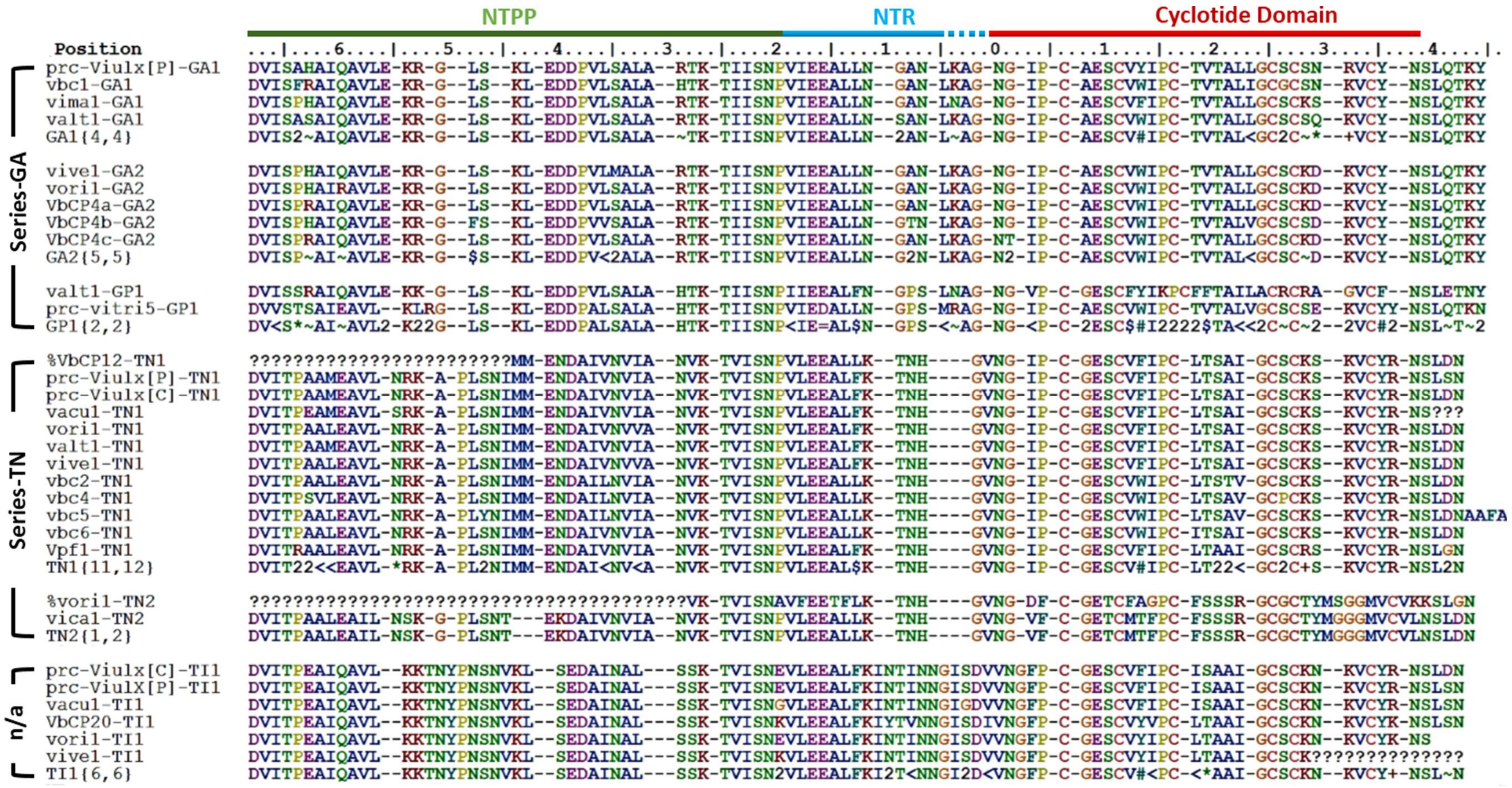
NTPP
NTR
Cyclotide Domain
Position ...|....6....|....5....|....4....|....3....|....2....|....1....|....0....|....1....|....2....|....3....|....4....|.
Series-GA
prc-Viulx[P]-GA1 DVISAHAIQAVLE-KR-G--LS--KL-EDDPVLSALA--RTK-TIISNPVIEEALLN--GAN-LKAG-NG-IP-C-AESCVYIPC-TVTALLGCSCSN--RVCY--NSLQTKY
vbc1-GA1 DVISFRAIQAVLE-KR-G--LS--KL-EDDPVLSALA--HTK-TIISNPVIEEALLN--GAN-LKAG-NG-IP-C-AESCVWIPC-TVTALIGCGCSN--KVCY--NSLQTKY
vima1-GA1 DVISPHAIQAVLE-KR-G--LS--KL-EDDPVLSALA--RTK-TIISNPVIEEALLN--GAN-LNAG-NG-IP-C-AESCVFIPC-TVTALLGCSCKS--KVCY--NSLQTKY
valt1-GA1 DVISASAIQAVLE-KR-G--LS--KL-EDDPVLSALA--RTK-TIISNPVIEEALLN--SAN-LKAG-NG-IP-C-AESCVWIPC-TVTALLGCSCSQ--KVCY--NSLQTKY
GA1{4,4} DVIS2~AIQAVLE-KR-G--LS--KL-EDDPVLSALA--~TK-TIISNPVIEEALLN--2AN-L~AG-NG-IP-C-AESCV#IPC-TVTAL<GC2C~*--+VCY--NSLQTKY
vive1-GA2 DVISPHAIQAVLE-KR-G--LS--KL-EDDPVLMALA--RTK-TIISNPVIEEALLN--GAN-LKAG-NG-IP-C-AESCVWIPC-TVTALLGCSCKD--KVCY--NSLQTKY
vori1-GA2 DVISPHAIRAVLE-KR-G--LS--KL-EDDPVLSALA--RTK-TIISNPVIEEALLN--GAN-LKAG-NG-IP-C-AESCVWIPC-TVTALLGCSCKD--KVCY--NSLQTKY
VbCP4a-GA2 DVISPRAIQAVLE-KR-G--LS--KL-EDDPVLSALA--RTK-TIISNPVIEEALLN--GAN-LKAG-NG-IP-C-AESCVWIPC-TVTALLGCSCKD--KVCY--NSLQTKY
VbCP4b-GA2 DVISPHAIQAVLE-KR-G--FS--KL-EDDPVVSALA--RTK-TIISNPVIEEALLN--GTN-LKAG-NG-IP-C-AESCVWIPC-TVTALVGCSCSD--KVCY--NSLQTKY
VbCP4c-GA2 DVISPRAIQAVLE-KR-G--LS--KL-EDDPVLSALA--RTK-TIISNPVIEEALLN--GAN-LKAG-NT-IP-C-AESCVWIPC-TVTALLGCSCKD--KVCY--NSLQTKY
GA2{5,5} DVISP~AI~AVLE-KR-G--$S--KL-EDDPV<2ALA--RTK-TIISNPVIEEALLN--G2N-LKAG-N2-IP-C-AESCVWIPC-TVTAL<GCSC~D--KVCY--NSLQTKY
valt1-GP1 DVISSRAIQAVLE-KK-G--LS--KL-EDDPVLSALA--HTK-TIISNPIIEEALFN--GPS-LNAG-NG-VP-C-GESCFYIKPCFFTAILACRCRA--GVCF--NSLETNY
prc-vitri5-GP1 DVVSTSAIEAVL--KLRG--LS--KL-EDDPALSALA--HTK-TIISNPVIEDALLN--GPS-MRAG-NG-IP-C-AESCVWIPC-TVTALVGCSCSE--KVCYY-NSLQTKN
GP1{2,2} DV<S*~AI~AVL2-K22G--LS--KL-EDDPALSALA--HTK-TIISNP<IE=AL$N--GPS-<~AG-NG-<P-C-2ESC$#I2222$TA<<2C~C~2--2VC#2-NSL~T~2
Series-TN
%VbCP12-TN1 ??????????????????????????MM-ENDAIVNVIA--NVK-TVISNPVLEEALLK--TNH----GVNG-IP-C-GESCVFIPC-LTSAI-GCSCKS--KVCYR-NSLDN
prc-Viulx[P]-TN1 DVITPAAMEAVL-NRK-A-PLSNIMM-ENDAIVNVIA--NVK-TVISNPVLEEALFK--TNH----GVNG-IP-C-GESCVFIPC-LTSAI-GCSCKS--KVCYR-NSLSN
prc-Viulx[C]-TN1 DVITPAAMEAVL-NRK-A-PLSNIMM-ENDAIVNVIA--NVK-TVISNPVLEEALFK--TNH----GVNG-IP-C-GESCVFIPC-LTSAI-GCSCKS--KVCYR-NSLDN
vacu1-TN1 DVITPEAMEAVL-SRK-A-PLSNIMM-ENDAIVNVIA--NVK-TVISNPVLEEALFK--TNH----GVNG-IP-C-GESCVFIPC-LTSAI-GCSCKS--KVCYR-NS???
vori1-TN1 DVITPAALEAVL-NRK-A-PLSNIMM-ENDAIVNVVA--NVK-TVISNPVLEEALFK--TNH----GVNG-IP-C-GESCVFIPC-LTSAI-GCSCKS--KVCYR-NSLDN
valt1-TN1 DVITPAAMEAVL-NRK-A-PLSNIMM-ENDAIVNVIA--NVK-TVISNPVLEEALFK--TNH----GVNG-IP-C-GESCVFIPC-LTSAI-GCSCKS--KVCYR-NSLDN
vive1-TN1 DVITPAALEAVL-NRK-A-PLSNIMM-ENDAIVNVVA--NVK-TVISNPVLEEALFK--TNH----GVNG-IP-C-GESCVFIPC-LTSAI-GCSCKS--KVCYR-NSLDN
vbc2-TN1 DVITPAALEAVL-NRK-A-PLSNIMM-ENDAILNVIA--NVK-TVISNPVLEEALLK--TNH----GVNG-IP-C-GESCVWIPC-LTSTV-GCSCKS--KVCYR-NSLDN
vbc4-TN1 DVITPSVLEAVL-NRK-A-PLSNIMM-ENDAILNVIA--NVK-TVISNPVLEEALLK--TNH----GVNG-IP-C-GESCVWIPC-LTSAV-GCPCKS--KVCYR-NSLDN
vbc5-TN1 DVITPAALEAVL-NRK-A-PLYNIMM-ENDAILNVIA--NVK-TVISNPVLEEALLK--TNH----GVNG-IP-C-GESCVWIPC-LTSAV-GCSCKS--KVCYR-NSLDNAAFA
vbc6-TN1 DVITPAALEAVL-NRK-A-PLSNIMM-ENDAIVNVIA--NVK-TVISNPVLEEALLK--TNH----GVNG-IP-C-GESCVWIPC-ITSAI-GCSCKS--KVCYR-NSLDN
Vpf1-TN1 DVITRAALEAVL-NRK-A-PLSNIMM-ENDAIVNVIA--NVK-TVISNPVLEEALFK--TNH----GVNG-IP-C-GESCVFIPC-LTAAI-GCSCRS--KVCYR-NSLGN
TN1{11,12} DVIT22<<EAVL-*RK-A-PL2NIMM-ENDAI<NV<A--NVK-TVISNPVLEEAL$K--TNH----GVNG-IP-C-GESCV#IPC-LT22<-GC2C+S--KVCYR-NSL2N
%vori1-TN2 ??VK-TVISNAVFEETFLK--TNH----GVNG-DF-C-GETCFAGPC-FSSSR-GCGCTYMSGGMVCVKKSLGN
vica1-TN2 DVITPAALEAIL-NSK-G-PLSNT---EKDAIVNVIA--NVK-TVISNPVLEEALLK--TNH----GVNG-VF-C-GETCMTFPC-FSSSR-GCGCTYMGGGMVCVLNSLDN
TN2{1,2} DVITPAALEAIL-NSK-G-PLSNT---EKDAIVNVIA--NVK-TVISNPVLEEALLK--TNH----GVNG-VF-C-GETCMTFPC-FSSSR-GCGCTYMGGGMVCVLNSLDN
n/a
prc-Viulx[C]-TI1 DVITPEAIQAVL--KKTNYPNSNVKL--SEDAINAL---SSK-TVISNEVLEEALFKINTINNGISDVVNGFP-C-GESCVFIPC-ISAAI-GCSCKN--KVCYR-NSLDN
prc-ViulX[P]-TI1 DVITPEAIQAVL--KKTNYPNSNVKL--SEDAINAL---SSK-TVISNEVLEEALFKINTINNGISDVVNGFP-C-GESCVFIPC-ISAAI-GCSCKN--KVCYR-NSLSN
vacu1-TI1 DVITPEAIQAVL--KKTNYPNSNVKL--SEDAINAL---SSK-TVISNGVLEEALFKINTINNGIGDVVNGFP-C-GESCVFIPC-ISAAI-GCSCKN--KVCYR-NSLSN
VbCP20-TI1 DVITPEAIQAVL--KKTNYPNSNVKL--SEDAINAL---SSK-TVISNKVLEEALFKIYTVNNGISDIVNGFP-C-GESCVYVPC-LTAAI-GCSCKN--KVCYK-NSLSN
vori1-TI1 DVITPEAIQAVL--KKTNYPNSNVKL--SEDAINAL---SSK-TVISNEVLEEALFKINTINNGISDVVNGFP-C-GESCVYIPC-LTAAI-GCSCKN--KVCYK-NS
vive1-TI1 DVITPEAIQAVL--KKTNYPNSNVKL--SEDAINAL---SSK-TVISNKVLEEALFKINTINNGISDVVNGFP-C-GESCVYIPC-LTAAI-GCSCK??????????????
TI1{6,6} DVITPEAIQAVL--KKTNYPNSNVKL--SEDAINAL---SSK-TVISN2VLEEALFKI2T<NNGI2D<VNGFP-C-GESCV#<PC-<*AAI-GCSCKN--KVCY+-NSL~N

Cont.

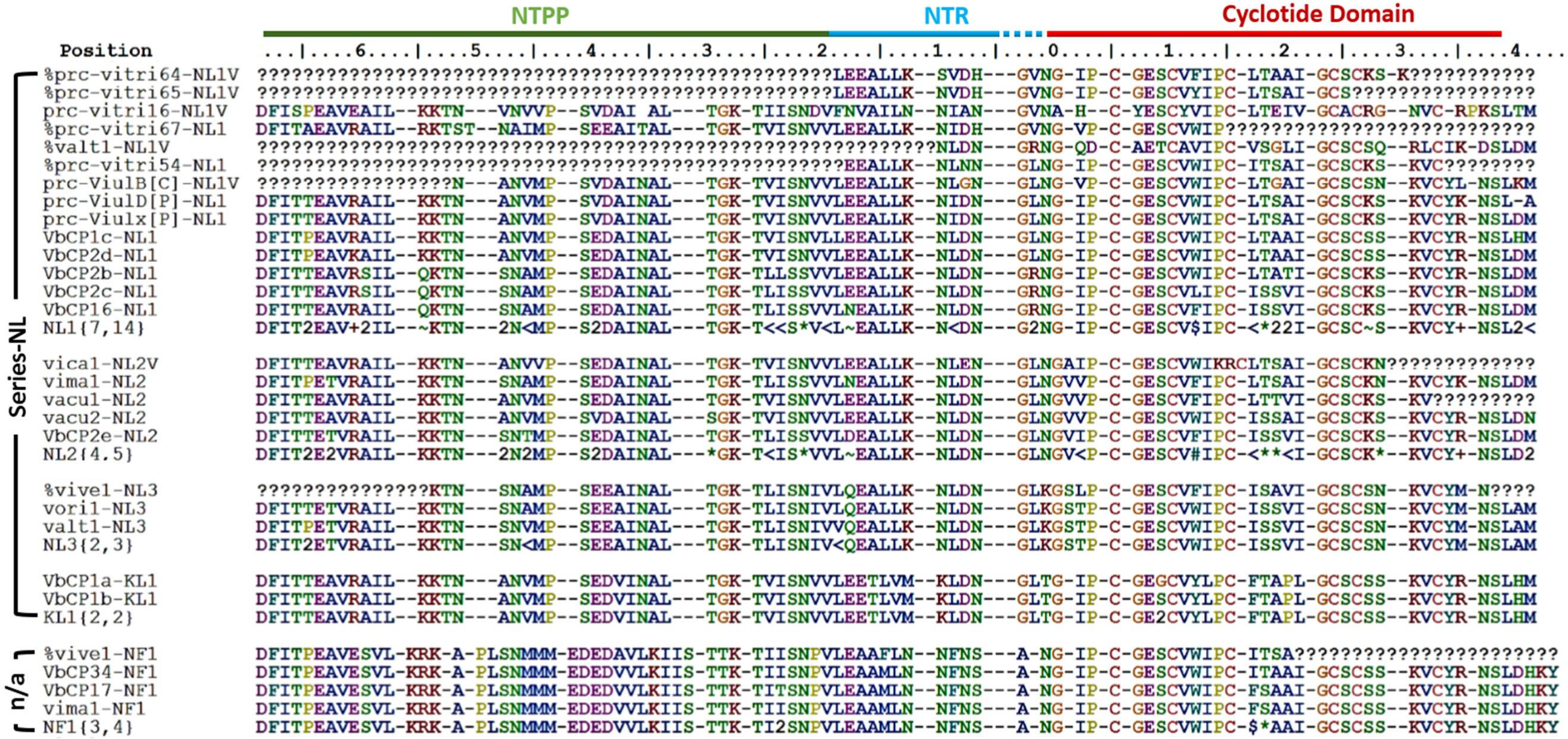

NTPP
NTR
Cyclotide Domain
Position ...|....6....|....5....|....4....|....3....|....2....|....1....|....0....|....1....|....2....|....3....|....4....
%prc-vitri64-NL1V ???LEEALLK--SVDH---GVNG-IP-C-GESCVFIPC-LTAAI-GCSCKS-K???????????
%prc-vitri65-NL1V ???LEEALLK--NVDH---GVNG-IP-C-GESCVYIPC-LTSAI-GCS????????????????
prc-vitri16-NL1V DFISPEAVEAIL--KKTN---VNVVP--SVDAI AL---TGK-TIISNDVFNVAILN--NIAN---GVNA-H--C-YESCYVIPC-LTEIV-GCACRG--NVC-RPKSLTM
%prc-vitri67-NL1 DFITAEAVRAIL--RKTST--NAIMP--SEEAITAL---TGK-TVISNVVLEEALLK--NIDH---GVNG-VP-C-GESCVWIP?????????????????????????????
%valt1-NL1V ??NLDN---GRNG-QD-C-AETCAVIPC-VSGLI-GCSCSQ--RLCIK-DSLDM
%prc-vitri54-NL1 ??EEALLK--NLNN---GLNG-IP-C-GESCVWIPC-ITSAI-GCSCKS--KVC????????
prc-ViulB[C]-NL1V ?????????????????N---ANVMP--SVDAINAL---TGK-TVISNVVLEEALLK--NLGN---GLNG-VP-C-GESCVWIPC-LTGAI-GCSCSN--KVCYL-NSLKM
prc-ViulD[P]-NL1 DFITTEAVRAIL--KKTN---ANVMP--SVDAINAL---TGK-TVISNVVLEEALLK--NIDN---GLNG-IP-C-GESCVWIPC-LTSAI-GCSCKS--KVCYK-NSL-A
prc-Viulx[P]-NL1 DFITTEAVRAIL--KKTN---ANVMP--SVDAINAL---TGK-TVISNVVLEEALLK--NIDN---GLNG-IP-C-GESCVWIPC-LTSAI-GCSCKS--KVCYR-NSLDM
VbCP1c-NL1 DFITPEAVRAIL--KKTN---ANVMP--SEDAINAL---TGK-TVISNVLLEEALLK--NLDN---GLNG-IP-C-GESCVWIPC-LTAAI-GCSCSS--KVCYR-NSLHM
VbCP2d-NL1 DFITPEAVKAIL--KKTN---ANVMP--SEDAINAL---TGK-TVISNVVLEEALLK--NLDN---GLNG-IP-C-GESCVWIPC-LTAAI-GCSCSS--KVCYR-NSLDM
VbCP2b-NL1 DFITTEAVRSIL--QKTN---SNAMP--SEDAINAL---TGK-TLLSSVVLEEALLK--NLDN---GRNG-IP-C-GESCVWIPC-LTATI-GCSCKS--KVCYR-NSLDM
VbCP2c-NL1 DFITTEAVRSIL--QKTN---SNAMP--SEDAINAL---TGK-TLISSVVLEEALLK--NLDN---GRNG-IP-C-GESCVLIPC-ISSVI-GCSCKS--KVCYR-NSLDM
VbCP16-NL1 DFITTEAVRAIL--QKTN---SNAMP--SEDAINAL---TGK-TLISSVVLNEALLK--NLDN---GRNG-IP-C-GESCVFIPC-ISSVI-GCSCKS--KVCYR-NSLDM
NL1{7,14} DFIT2EAV+2IL--~KTN---2N<MP--S2DAINAL---TGK-T<<S*V<L~EALLK--N<DN---G2NG-IP-C-GESCV$IPC-<*22I-GCSC~S--KVCY+-NSL2<
vica1-NL2V DFITTEAVRAIL--KKTN---ANVVP--SEDAINAL---TGK-TVISNVVLEEALLK--NLEN---GLNGAIP-C-GESCVWIKRCLTSAI-GCSCKN?????????????
vima1-NL2 DFITPETVRAIL--KKTN---SNAMP--SEDAINAL---TGK-TLISSVVLNEALLK--NLDN---GLNGVVP-C-GESCVFIPC-LTSAI-GCSCKN--KVCYK-NSLDM
vacu1-NL2 DFITTEAVRAIL--KKTN---ANVMP--SEDAINAL---TGK-TVISNVVLEEALLK--NLDN---GLNGVVP-C-GESCVFIPC-LTTVI-GCSCKS--KV?????????
vacu2-NL2 DFITTEAVRAIL--KKTN---ANVMP--SVDAINAL---SGK-TVISNVVLEEALLK--NLDN---GLNGVVP-C-GESCVWIPC-ISSAI-GCSCKS--KVCYR-NSLDN
VbCP2e-NL2 DFITTETVRAIL--KKTN---SNTMP--SEDAINAL---TGK-TLISSVVLDEALLK--NLDN---GLNGVIP-C-GESCVFIPC-ISSVI-GCSCKS--KVCYR-NSLDM
NL2{4.5} DFIT2E2VRAIL--KKTN---2N2MP--S2DAINAL---*GK-T<IS*VVL~EALLK--NLDN---GLNGV<P-C-GESCV#IPC-<**<I-GCSCK*--KVCY+-NSLD2
%vive1-NL3 ??????????????KTN---SNAMP--SEEAINAL---TGK-TLISNIVLQEALLK--NLDN---GLKGSLP-C-GESCVFIPC-ISAVI-GCSCSN--KVCYM-N????
vori1-NL3 DFITTETVRAIL--KKTN---SNAMP--SEEAINAL---TGK-TLISNIVLQEALLK--NLDN---GLKGSTP-C-GESCVWIPC-ISSVI-GCSCSN--KVCYM-NSLAM
valt1-NL3 DFITPETVRAIL--KKTN---SNVMP--SEEAINAL---TGK-TLISNIVVQEALLK--NLDN---GLKGSTP-C-GESCVWIPC-ISSVI-GCSCSN--KVCYM-NSLAM
NL3{2,3} DFIT2ETVRAIL--KKTN---SN<MP--SEEAINAL---TGK-TLISNIV<QEALLK--NLDN---GLKGSTP-C-GESCVWIPC-ISSVI-GCSCSN--KVCYM-NSLAM
VbCP1a-KL1 DFITTEAVRAIL--KKTN---ANVMP--SEDVINAL---TGK-TVISNVVLEETLVM--KLDN---GLTG-IP-C-GEGCVYLPC-FTAPL-GCSCSS--KVCYR-NSLHM
VbCP1b-KL1 DFITTEAVRAIL--KKTN---ANVMP--SEDVINAL---TGK-TVISNVVLEETLVM--KLDN---GLTG-IP-C-GESCVYLPC-FTAPL-GCSCSS--KVCYR-NSLHM
KL1{2,2} DFITTEAVRAIL--KKTN---ANVMP--SEDVINAL---TGK-TVISNVVLEETLVM--KLDN---GLTG-IP-C-GE2CVYLPC-FTAPL-GCSCSS--KVCYR-NSLHM
%vive1-NF1 DFITPEAVESVL-KRK-A-PLSNMMM-EDEDAVLKIIS-TTK-TIISNPVLEAAFLN--NFNS---A-NG-IP-C-GESCVWIPC-ITSA?????????????????????????
VbCP34-NF1 DFITPEAVESVL-KRK-A-PLSNMMM-EDEDVVLKIIS-TTK-TIISNPVLEAAMLN--NFNS---A-NG-IP-C-GESCVWIPC-ITAAI-GCSCSS--KVCYR-NSLDHKY
VbCP17-NF1 DFITPEAVESVL-KRK-A-PLSNMMM-EDEDVVLKIIS-TTK-TITSNPVLEAAMLN--NFNS---A-NG-IP-C-GESCVWIPC-FSAAI-GCSCSS--KVCYR-NSLDHKY
vima1-NF1 DFITPEAVESVL-KRK-A-PLSNMMM-EDEDVVLKIIS-TTK-TIISNPVLEAAMLN--NFNS---A-NG-IP-C-GESCVWIPC-FSAAI-GCSCSS--KVCYR-NSLDHKY
NF1{3,4} DFITPEAVESVL-KRK-A-PLSNMMM-EDEDVVLKIIS-TTK-TI2SNPVLEAAMLN--NFNS---A-NG-IP-C-GESCVWIPC-$*AAI-GCSCSS--KVCYR-NSLDHKY
Series-NL
n/a

Cont.

```
                                           NTPP                                            NTR                    Cyclotide Domain
Position              ...|....6....|....5....|....4....|....3....|....2....|....1....|....0....|....1....|....2....|....3....|....4....

n/a
prc-vitri17-HQ1       DVISAEAVQTIL---KMN-PSSNSIP---D-INEIV---KMK-TVVSSSVLDPFILK--HQGGGSHGGSDDQVAC-GESCAMTPC-FMHVV-GCVCSQ--KVCYRG
%prc-vitri66-HQ1      ????????????????????????????????????????????????VLDHDAFILK--HH-GGSHG-SDDQMPC-GESCVYIPC-FTRVV-GCACSE--KVCYKG
HQ1{1,2}              DVISAEAVQTIL---KMN-PSSNSIP---D-INEIV---KMK-TVVSSSVLDPFILK--HQGGGSHGGSDDQVAC-GESCAMTPC-FMHVV-GCVCSQ--KVCYRG

vacu1-NK1             ????????????????????NSN-GML-DEKSINAL---TRK-SIISKQLFEEAFLKDSNKIENLGGTGFTEP-C-GETCRILAC--GIP--GCQC-Y-ARICYW-NSLA
vima1-NK1             ????????????????????NSN-GML-DEKSITAL---TSK-TIISKQLYEEAFLKDSNRIENLGGTGFTEP-C-GENCRILAC--GIP--GCQC-Y-KRVCYW-NSLA
NK1{2,2}              ????????????????????NSN-GML-DEKSI*AL---T~K-*IISKQL#EEAFLKDSN+IENLGGTGFTEP-C-GE*CRILAC--GIP--GCQC-Y-2R<CYW-NSLA

Series-RS
%prc-vitri98-RS1V     ????????????????????????MLS--EDAF------TSKIVVADNLVLEEAL-KMMTRRRDDEKLGG-IP-C-GETCVFSGC-YSVTF-GCACEK--RVCYK-NSL-PS
prc-vitri60-RS1V      DLITPVAIQAIL-QKTSS-DFNF-MLSQSSEV-------TSKIVVADNLVLEEAL-LMMRRS--DEKLGG-IP-C-GETCIFGRC-HTGII-GCACEK--YMCCK-NSL-PS
%vive1-RS1            ??????????????????????????????????????????KIVIADNLVLEKALGMMIR-NNIK--LDG-IP-C-AETCIWRPC-ATAII-GCYCEN--YMCYK-NSL-AT
vacu1-RS1             DVITPEAIQAILDQKKTN-PNSNYMFSDSEDAVNAL---TGKIVIADNLVLEEAL-KMMRSNNIK--LDG-IP-C-AETCLWRPC-HTAII-GCSCEY--NFCYK-NSLLAN
prc-ViulL[C]-RS1      DVITPEAIQAILDQKKTN-PNSNYMLSDSEDAVNAL---TGKIVIADNLFLEEAL-KMMRSNNVK--LDG-IP-C-AETCLWRPC-RTAIM-GCSCEY--NFCYK-NSLLAN
RS1{2,5}              DVITPEAIQAILDQKKTN-PNSNYM$SDSEDAVNAL---TGKIVIADNL$LEEAL-KMMRSNN<K--LDG-IP-C-AETCLWRPC-~TAI<-GCSCEY--NFCYK-NSLLAN

vica1-RS2V            DVITPEAIQAILHQKKTN-PNSNYMLSDSEDAVNAL---TGKIVIAGNLVLEEAL-KMMRSNNVK--LDG-IH-C-EETCFWRPC-HTAIV-GCSCEY--NICFK-NSLLAN
%VbCP13-RS2           ????????????????????????MLSDSEDAVNAL---TGKTVIADNLFLEEAL-RMMRRNNVK--LDG-IH-C-AETCLWGTC-RTAYI-GCSCEN--KICYK-NSLLAN
%VbCP22-RS2           ????????????????????????MLSDSEDAVNAL---TGKIVIADNLVLEEAL-RMMRSDNVK--LDG-IH-C-AETCIWGTC-RTAII-GCSCEN--RICYK-NSLLAN
%VbCP38-RS2           ????????????????????????MLSDSEDAVNAL-----KIVIADNLFLEEAL-RMMRSNNVK--LDG-IH-C-AETCFWGTC-RTAYI-GCSCEN--RICYK-NSLLAN
%vive1-RS2            DVITPEAIQAILDQKKTN-PNSNYMLSGSEDAVNAL---TGKIVIADNLVLEEAL-KMMRSNNVK--LDG-IH-C-AETCFWGKC-HTAII-GCSCEN--KICYK-NSLLAN
%vive2-RS2            DVITPEAIQAILDQKKTN-PNSNYMLSDSEDAVNAL---TGKIVIADNLVLEEAL-RMMRSNNVK--LDG-IH-C-AETCFWGKC-HTAII-GCSCEN--KICYK-NSLLAN
vima1-RS2             DVITPEAIQAILDQKKTN-PNSNYMLSDSEDVVNAL---TGKIVIADNLFLEEAL-RMMRSNSVK--LDG-IH-C-AETCLWGTC-RTAYI-GCSCEN--KICYK-NSLLAN
vori1-RS2             DVITPEAIQAILHQKKTN-PNSNYMLSDSEDAVNAL---TGKIVIADNLVLEEAL-RMMRSNNVK--LDG-IH-C-AETCFWGKC-YTAIT-GCSCEN--KICYK-NSLLAN
valt1-RS2             DVITPEAIQAILDQKKTN-PNSNYMLSDSEDAVNAL---TGKIVIADKLFLEEAL-RMMRSNNVK--LDG-IH-C-AETCFWGTC-RTAYI-GCSCEN--KICYK-NSLLAN
valt2-RS2             DVITPEAIQAILDQKKTN-PNSNYMLSDSEDAVNAL---TGKIVIADKLFLEEAL-RMMRSNNVK--LDG-IH-C-AETCFWGTC-RTAYI-GCSCEN--KICYK-NSLLAN
RS2{4,10}             DVITPEAIQAIL~QKKTN-PNSNYMLSDSED<VNAL---TGKIVIAD~L$LEEAL-RMMRSN*VK--LDG-IH-C-AETC$WG~C-2TA$2-GCSCEN--KICYK-NSLLAN

n/a
%valt1-DI1            ?????????????????????????????????????????????????VFE-SFL--DIHN-LEG-E--VV-C-GPTCEFGPVNCGINP-SCRCNN--RVCVY-NSLSG
vica1-DI1             DIISGDQVVAML---KSG---------EDLESMNAML--K-R-TAVVSGEVFE-SFL--DTHN-LEG----AV-C-GPTCQFGPVNCGINP-SCRCNN--KVCVM-NSLSG
vima1-DI1             DIISGHQVVAML---KSG---------QDLESMNAML--K-R-TAVVSGEVFE-SFF--DIHN-LEG-E--VV-C-GPSCMYGPVNCGINP-SCKCNN--KFCVM-NSLRG
vima2-DI1             DIISGEQVVAML---KRG---------EGLESMNAML--K-R-TAVVTGEVLE-SFL--DTHNLLKG-DG-VQ-C-GPTCRFGPVNCGINP-KCNCRN--TLCVW-DPLEY
VbCP21-DI1            DIISGEQVVAML---KRG---------EDLESINAML--K-R-TAIVTGEVFE-SFL--EAHNLLKG-DG-VQ-C-GPTCRFGPVNCGINP-RCNCRN--TRCVW-DPLEY
DI1{4,5}              DIISG~QVVAML---K~G---------~2LES<NAML--K-R-TA<V*GEV$E-SF$--=2HN2L~G-22-<2-C-GP*C2#GPVNCGINP-~C~C~N--~2CV$-~2L~2
```

Cont.

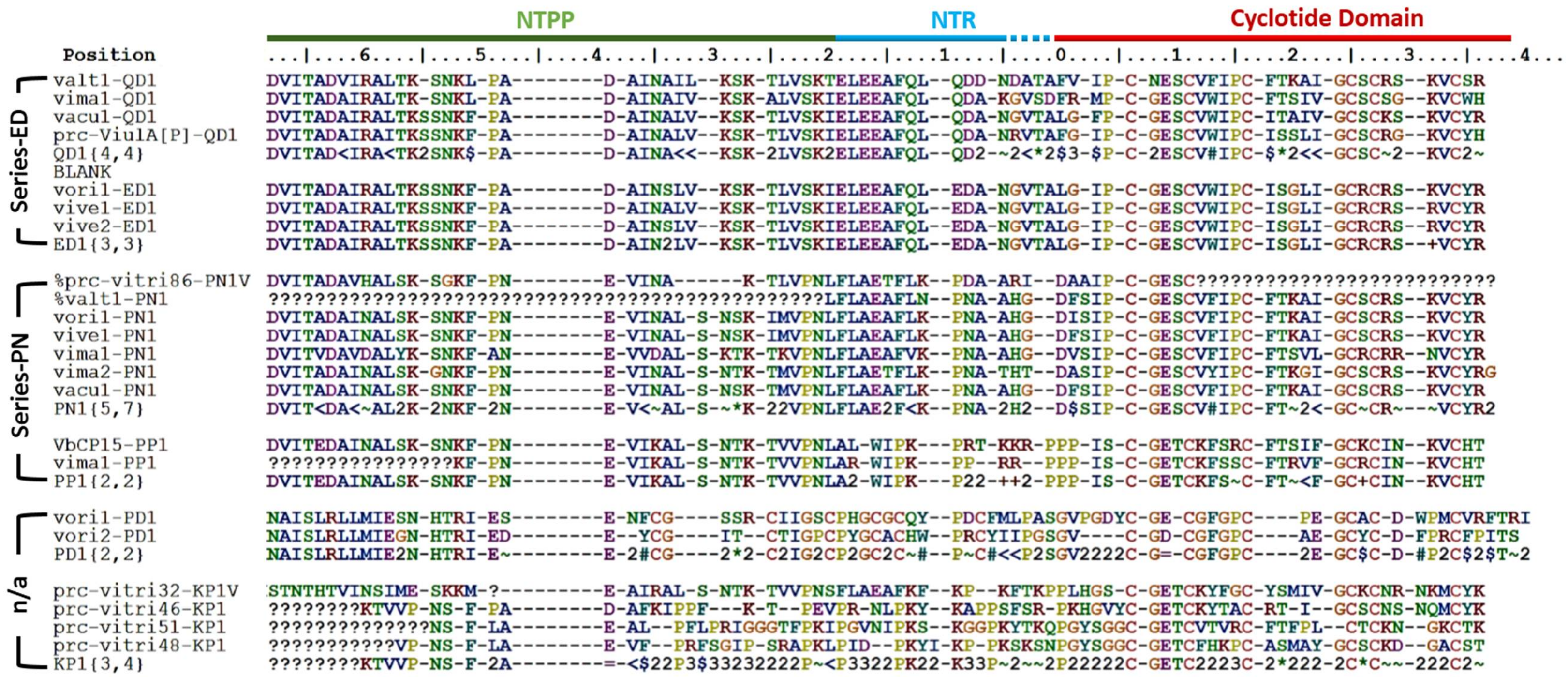
NTPP
NTR
Cyclotide Domain
Position
Series-ED
Series-PN
n/a
valt1-QD1
vima1-QD1
vacu1-QD1
prc-Viu1A[P]-QD1
QD1{4,4}
BLANK
vori1-ED1
vive1-ED1
vive2-ED1
ED1{3,3}
%prc-vitri86-PN1V
%valt1-PN1
vori1-PN1
vive1-PN1
vima1-PN1
vima2-PN1
vacu1-PN1
PN1{5,7}
VbCP15-PP1
vima1-PP1
PP1{2,2}
vori1-PD1
vori2-PD1
PD1{2,2}
prc-vitri32-KP1V
prc-vitri46-KP1
prc-vitri51-KP1
prc-vitri48-KP1
KP1{3,4}
