# Supplementary material for: Cyclotide Evolution: Insights from the Analyses of Their Precursor Sequences, Structures and Distribution in Violets (Viola)
Source: Front Plant Sci. 2017 Dec 18;8:2058. doi: 10.3389/fpls.2017.02058 (PMC5741643; doi:10.3389/fpls.2017.02058)

Supplementary Figure 2. Multiple sequence alignment of precursors unclassified into molecular species

We could not assign the 12.0% (=34/283) precursors into molecular species. Out of the 34 precursors, 10 precursors are grouped into three groups with the sequence signatures from both NTR and cyclotide domains: i) vacu1-FA2U, vima1-FA2U and valt1-FA2U, ii) VbCP30-YY3U, VbCP28-YY3U, VbCP32-YY3U, VbCP33-YY3U and VbCP35-YY3U, and iii) prc-vitri34-HV1U and prc-vitri92-HV1U (panel A). Based on the sequence signature in NTR [-9, -8], the first two groups are belong to Möbius lineage, and the last group is belong to bracelet lineage. Because all of those precursors lack the NTPP sequences from the transcriptomic assay, we excluded these sequences from the classification into molecular species.

Out of the 34 precursors, the seven precursor sequences were also excluded from the classification, because these sequences has lone unique sequence signatures. However, those sequences satisfy the criteria as bracelet linear with the sequence signature in NTPP [-56, -38] (panel B).

The rest 17 precursor sequences are grouped into the lineages based on the sequence signature of NTR [-9, -8]. The precursors belong to Möbius and bracelet lineages are listed in the panel C and the panel D, respectively. Out of the 17 precursors, we could not assign 14 precursor sequences into molecular species, because these precursors either lack the NTPP sequences from the transcriptomic assay or have lone unique sequence signatures. Only three precursors 1.1% (=3/283) have complete sequences, but not to meet the criteria classifying lineage with NTPP domain sequences. These precursor sequences are: i) vica1-FA3U whose NTPP and NTR domain have large sequence variation, ii) vori1-YY4U whose NTPP domain has sequence deletion, iii) vima1-YV1 whose NTR [-9, -8] signature is Möbius lineage, but contain sequence insertion in NTPP [-56, -38] of bracelet lineage.

Unclassified precurosors

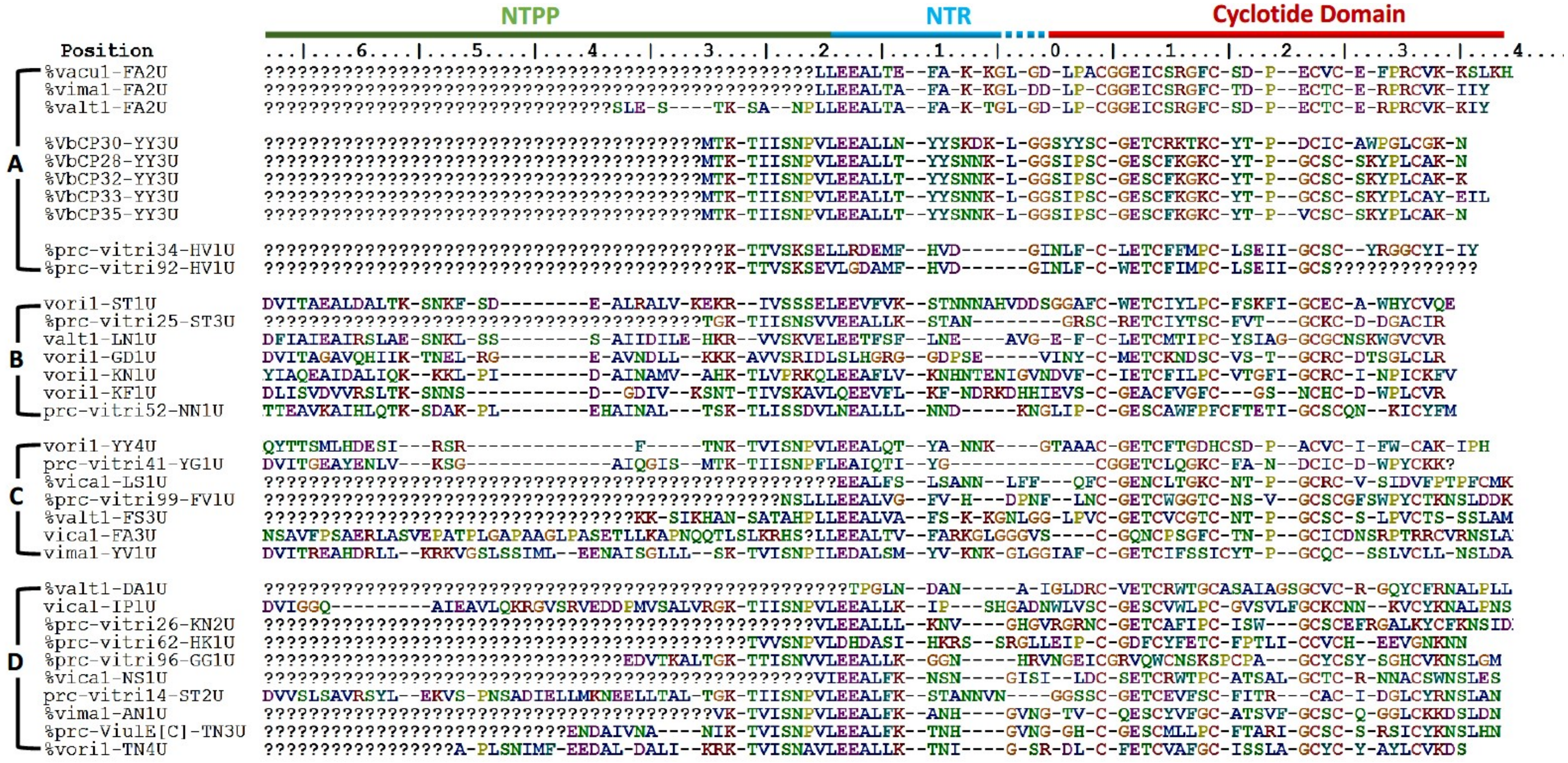

Supplement: Supplementary file 11 [file Image2.PDF]
