# Supplementary figures and images for: Cyclotide Evolution: Insights from the Analyses of Their Precursor Sequences, Structures and Distribution in Violets (Viola)
Source: Front Plant Sci. 2017 Dec 18;8:2058. doi: 10.3389/fpls.2017.02058 (PMC5741643; doi:10.3389/fpls.2017.02058)

A

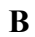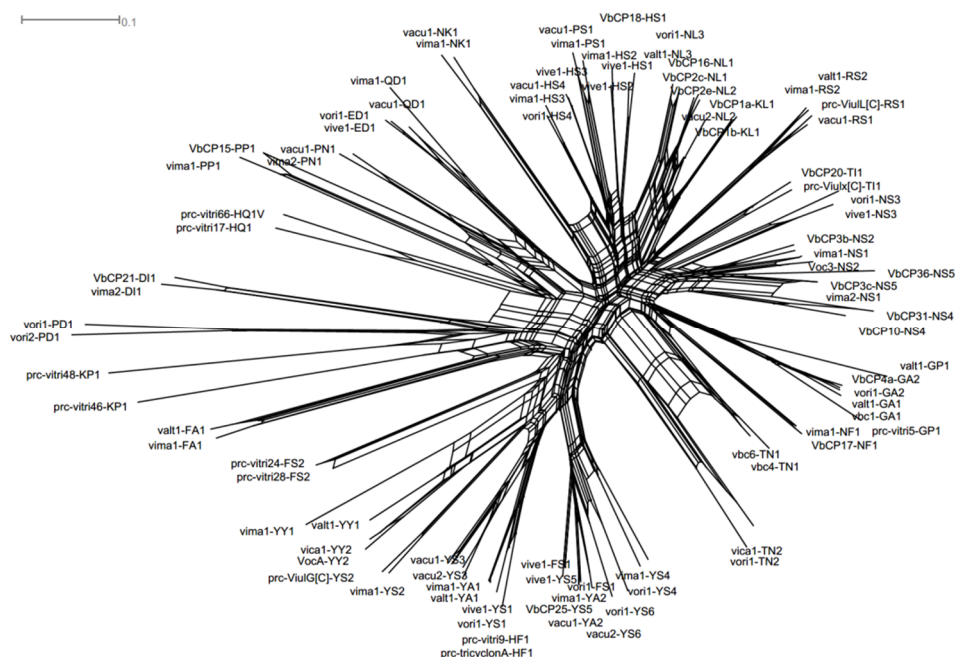

Supplement: Supplementary file 12 [file Image3.PDF]
