# Supplementary material for: Cyclotide Evolution: Insights from the Analyses of Their Precursor Sequences, Structures and Distribution in Violets (Viola)
Source: Front Plant Sci. 2017 Dec 18;8:2058. doi: 10.3389/fpls.2017.02058 (PMC5741643; doi:10.3389/fpls.2017.02058)
Supplement: Supplementary file 13 [file Image4.PDF]

Supplementary Figure 4. The distribution of molecular species and series found across *Viola* sections

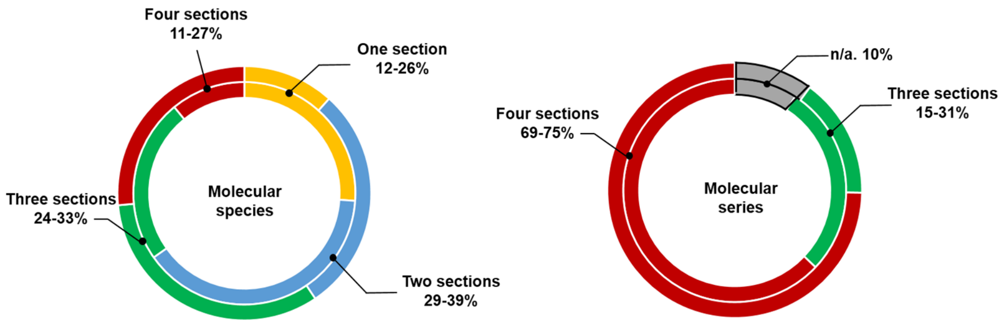

A. Distribution of molecular species

Number of sections	Complete sequences only	Complete sequences and partial sequences
1	YA1{P}, HF1{M}, YY1{P}, FS2{M}, HS3{P}, PS1{P}, NS1{P}, NS4{P}, NS5{P}, TN2{C}, KL1{P}, PP1{P}, NF1{P}, HQ1{M}, PD1{C}, KP1{M}	YY1{P}, FS2{M}, HS3{P}, NS1{P}, NS4{P}, TN2{C}, KL1{P}, PP1{P}, NF1{P}, HQ1{M}, PD1{C}, KP1{M}
2	YS4{C,P}, YS5{P,V}, YA2{P,V}, FS1{C,P}, YS3{C,V}, YS2{P,V}, YS1{C,P}, YY2{C,V}, HS1{P,V}, HS2{P,V}, GA1{P,V}, GA2{C,P}, GP1{P,M}, NL1{P,V}, NL3{C,P}, RS1{P,V}, RS2{C,P}, QD1{P,V}, ED1{C,P}, NK1{P,V}, DI1{C,P}	YS4{C,P}, YA2{P,V}, YA1{P,M}, YS2{P,V}, YS1{C,P}, HF1{P,M}, YY2{C,V}, PS1{P,V}, GA1{P,V}, GA2{C,P}, GP1{P,M}, NL3{C,P}, RS2{C,P}, QD1{P,V}, ED1{C,P}, NK1{P,V}, DI1{C,P}
3	YS6{C,V,M}, HS4{C,P,V}, NS2{C,P,V}, NS3{C,P,V}, TN1{C,P,V}, NL2{C,P,V}, PN1{C,P,V}, TI1{C,P,V}	YS6{C,V,M}, YS3{C,V,M}, HS1{C,P,V}, HS2{P,V,M}, NS3{C,P,V}, NS5{C,P,M}, TN1{C,P,V}, NL1{P,V,M}, NL2{C,P,V}, RS1{P,V,M}, TI1{C,P,V}
4	FA1{C,P,V,M}	YS5{C,P,V,M}, FA1{C,P,V,M}, HS4{C,P,V,M}, NS2{C,P,V,M}, PN1{C,P,V,M}

B. Distribution of molecular series

Number of sections	Complete sequences only	Complete sequences and partial sequences
1	n/a	n/a
2	n/a	n/a
3	HF{C,P,M}, YY{C,P,V}, TN{C,P,V} ED{C,P,V}	YA{C,P,V}, HF{C,P,M}, YY{P,C,V}, HS{C,P,V}, NS{C,P,V}, TN{C,P,V}, NL{C,P,V}, RS{C,P,V}, ED{C,P,V}, PN{C,P,V}
4	YS{C,P,V,M}, YA{C,P,V,M}, FA{C,P,V,M}, HS{C,P,V,M}, NS{C,P,V,M}, GA{C,P,V,M}, NL{C,P,V,M}, RS{C,P,V,M}, PN{C,P,V,M}	YS{C,P,V,M}, FA{C,P,V,M}, GA{C,P,V,M}

List of molecular species found across *Viola* sections. The multiple letters in the bracket "{ }" shows the name of *Viola* sections where the molecular species were found. The letter "P" represent for *Plagiostigma,* "V" for *Viola*, "C" for *Chamaemelanium* and "M" for *Melanium.*
