# Supplementary material for: Cyclotide Evolution: Insights from the Analyses of Their Precursor Sequences, Structures and Distribution in Violets (Viola)
Source: Front Plant Sci. 2017 Dec 18;8:2058. doi: 10.3389/fpls.2017.02058 (PMC5741643; doi:10.3389/fpls.2017.02058)

Supplementary Figure 5. Multiple sequence alignment of precursor between *Viola* and the other Violaceae genera

Some of the precursor sequences have sequence homology, although they are found different genera (*i.e. Viola* and the other Violaceae genera). The sequence homology is found with the different sequence regions between lineages: NTPP [-56, -38] and NTR [-9, -8] in bracelet lineage, and the NTR [-9, -8] in Möbius lineage. In bracelet lineage, such examples are found in the molecular species-NS2, HS3, QD1 and GA1: i) in NS2, the precursors from *Viola* (*i.e.* vacu1-NS2, VbCP3b-NS2 and VbCP3d-NS2) and the precursors (*i.e.* Mra15 and Mra17) from *Melicytus*, ii) in HS3, the precursor from *Viola* (*i.e.* vere1-HS3) and the precursors from *Pigea* (*i.e.* prc-Hyfl L, prc-Hyfl D and prc-Hyfl K), iii) in QD1, the precursors from *Viola* (*i.e.* valt1-QD1 and vacu1-QD1) and the precursor from *Melicytus* (*i.e.* Mra13) and iv) in GA1, the precursor from *Viola* (*i.e.* vbc1-GA1) and the precursor (Gpc3) from genus *Gleospermum*. Also, in Mobius lineage, the molecular species-YS2 is found with sequence homology. These are the precursors from *Viola* (*i.e.* vacu-YS2 and vima-YA2) and the precursors from *Melicytus* (*i.e.* Mra24 and Mra26).

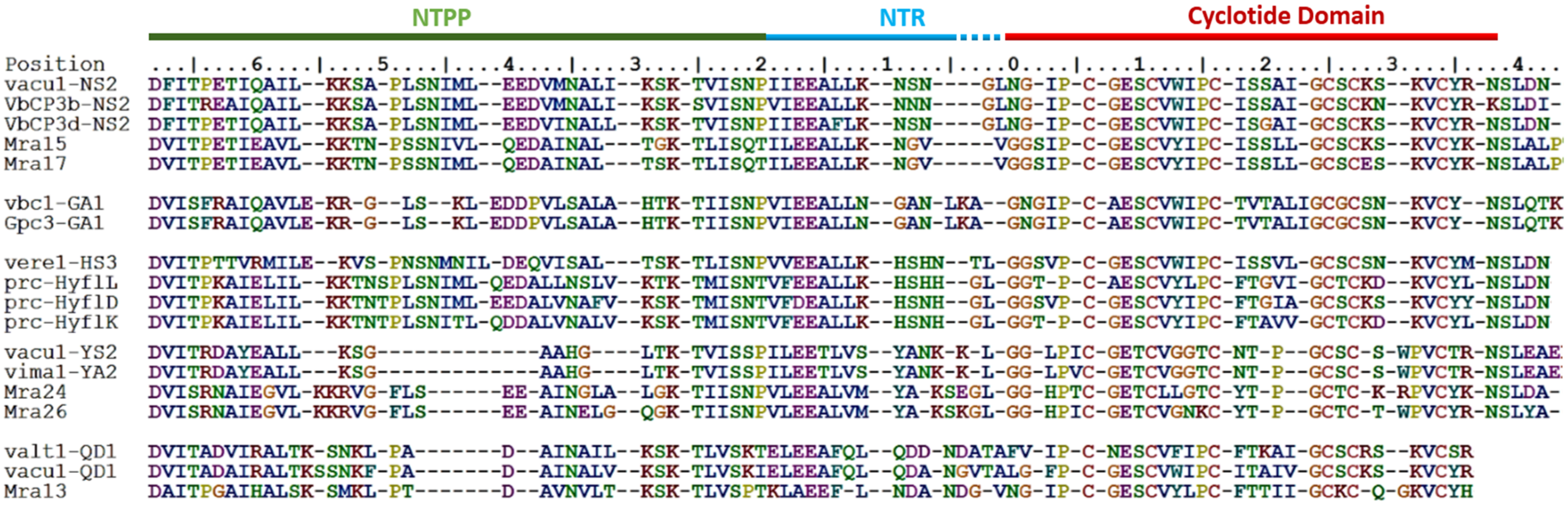

Supplement: Supplementary file 14 [file Image5.PDF]
