# Supplementary material for: Cyclotide Evolution: Insights from the Analyses of Their Precursor Sequences, Structures and Distribution in Violets (Viola)
Source: Front Plant Sci. 2017 Dec 18;8:2058. doi: 10.3389/fpls.2017.02058 (PMC5741643; doi:10.3389/fpls.2017.02058)
Supplement: Supplementary file 15 [file Image6.PDF]

Supplementary Figure 6. Hydrophobicity of the cyclotides from transcriptome

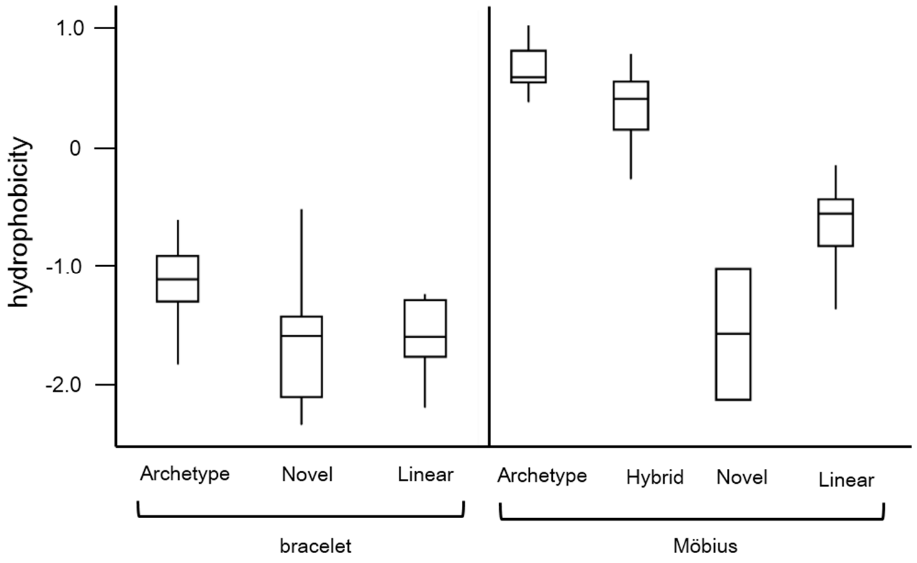

The boxes show the range between the first and third quartiles, and the upper and lower error bars represent the maximum and minimum hydrophobicity of the cyclotides, respectively.
